# Exploring the biotechnological potential of terrestrial hot spring microbiomes for CO_2_ utilisation

**DOI:** 10.1186/s40793-026-00875-x

**Published:** 2026-03-11

**Authors:** Christopher E. Stead, Leanne Walker, Carla Greco, Toni Galloway, Claire R Cousins, Franziska Nagel, Rainer Breitling, Eriko Takano, Snaedís Huld Björnsdóttir, Sophie L Nixon

**Affiliations:** 1 Manchester Institute of Biotechnology, University of Manchester, Manchester, UK; 2 Basecamp Research, London, UK; 3 School of Earth and Environmental Sciences, University of St Andrews, St Andrews, UK; 4 Bioinformatics Institute (BII), Agency for Science, Technology and Research (A*STAR), Singapore, Singapore; 5 Singapore Integrative Biosystems and Engineering Research (SIBER) Strategic Research Translational Trust (SRTT), Singapore, Singapore; 6 School of Engineering and Natural Sciences, University of Iceland, Reykjavik, Iceland

**Keywords:** Hot spring microbiomes, Comparative metagenomics, CO_2_ utilisation, Chemolithoautotrophy, Biosynthetic gene clusters, Microbiome engineering

## Abstract

**Background:**

Terrestrial hot springs are extreme environments shaped by geothermal heat, geogenic gases and extremes of pH and temperatures. Their gas fluxes, which include CO_2_, CO, H_2_S and SO_2_, mirror the chemical composition of CO_2_-rich waste streams. Microbial communities inhabiting these environments are typically thermotolerant or thermophilic and sustained by CO_2_ fixation and chemolithotrophic metabolism. Such communities may therefore provide a natural starting point for developing *ex-situ*, consortium-based biotechnologies capable of operating under elevated temperatures and chemically harsh conditions. Here, we assess the metabolic capabilities of hot spring microbiomes systematically through a biotechnological lens.

**Results:**

We conducted comparative analysis of 73 worldwide hot spring metagenomes, spanning a wide range of environmental conditions (pH 1.5–10.0, temperatures 25–98 °C). By taking a gene-centric approach to whole communities, we show that hot spring microbiomes ubiquitously encoded carbon fixation pathways and biosynthetic genes (and gene clusters) for the synthesis of value-added products, regardless of geographical location and pH-temperature conditions. Candidate value-added products include platform chemicals such as acetone, lactic acid, and 1,2-propanediol, as well as high-value biomolecules including B vitamins and alginate.

**Conclusions:**

This first biotechnology-focused assessment of hot spring microbiomes demonstrates that these communities encode the genomic potential to support novel, *ex situ* microbial platforms for upgrading CO_2_ and transforming chemically complex gas mixtures.

**Significance:**

Industrial CO_2_ waste streams pose both an environmental challenge and an unutilised resource. Harnessing microbial consortia to valorise CO_2_, through a circular bioeconomy, remains underexplored and could offer an alternative to energy-intensive chemical methods. By reanalysing predominantly publicly available metagenomic data, we demonstrate how hot spring microbiomes can be mined for traits pre-adapted to CO_2_-rich, high-temperature, and chemically extreme conditions. In doing so, we provide proof-of-concept for their future biotechnological application and establish a blueprint for other microbiome-scale bioprospecting surveys.

**Supplementary Information:**

The online version contains supplementary material available at 10.1186/s40793-026-00875-x.

## Introduction

Industrial processes associated with the production of steel, cement and iron release waste gases rich in CO_2_, along with heat. These waste streams represent both an environmental challenge and a potential resource. Circular biotechnological approaches harness the biocatalytic properties of organisms to sequester and upgrade waste industrial CO_2_ into value-added products. These biotechnologies offer a low energy and cost-effective alternative to existing chemical transformations. We hypothesise that microbial communities inhabiting terrestrial hot springs provide a promising natural basis for new biotechnologies. These communities are pre-adapted to fix CO_2_ efficiently and independent of light [1], transform CO_2_ through interconnected cross-feeding and trophic interactions [2], detoxify oxidised and reduced sulfur species common in industrial emissions, and thrive under elevated temperatures. In this study, we present the first biotechnology-focused comparative analysis of hot spring metagenomes, assessing how pH and temperature constrain microbial diversity and the utilisation of CO_2_, CO, and other common industrial waste gases. In parallel, we demonstrate the metabolic potential of these communities for the biosynthesis of value-added products. By linking community structure to functional potential, we provide a blueprint for harnessing hot spring microbiomes as a foundation for future microbiome engineering and CO_2_ utilisation strategies.

Realising such strategies requires overcoming the specific challenges posed by industrial waste gas compositions. Developing CO_2_-utilising biotechnologies requires tackling not only CO_2_ but the broader physicochemical environment of industrial waste streams. These gases often contain impurities including CO, H_2_, SO_2_, and NOₓ. For instance, syngas from steel mills contains approximately 23.9% CO, 20.5% CO_2_, and 6.5% H_2_ [3], while cement and coal waste gases are enriched in SO_2_ (25–200 mg/m³) and NOₓ (250–300 mg/m³) at high temperatures (80–165 °C) [4, 5]. Effective CO_2_ valorisation strategies must therefore operate under elevated temperatures and chemically extreme conditions. Microalgal systems that combine CO_2_, NO_x_, and SO_x_ to produce lipid-rich biomass have shown promise [6–10], but are limited by low pH inhibition from dissolved SO_x_ and NO_x_ [11], poor thermotolerance under industrial temperatures [12], and the need for light. Non-photosynthetic approaches using single strains of acetogens to consume CO/CO_2_ via the Wood-Ljungdahl (WL) pathway represent another demonstrated route, yielding products such as acetate, ethanol, lactate, butanol and 2,3-butanediol [13]. Increasingly, co-culture systems demonstrate advantages in overcoming metabolic limitations that are common to single-strain approaches [14, 15]. However, microbiome-based approaches remain underexplored for industrial applications, despite increasing acknowledgement of their promise [16–19].

Historically, hot spring communities have been an important source of biotechnological innovation, primarily through the discovery of thermostable enzymes [20–22] and the isolation of individual microbial strains [23–26]. In contrast, the use of these communities as integrated consortia for the development of *ex situ* CO_2_-utilisation strategies remains underexplored. Here, we define terrestrial hot springs as surface environments where fluids of meteoritic and / or subsurface origin interact with geothermal heat and geogenic gases, resulting in surface waters with elevated temperatures relative to their surroundings (25–98 °C in this study). These systems are naturally CO_2_-rich and are often subject to fluxes of other gases similar to industrial waste streams, including CO, H_2_, H_2_S, and SO_2_ [27]. Across geothermal regions such as Yellowstone National Park (USA), Kamchatka Peninsula (Russia), North Island of New Zealand, East African Rift, and Iceland, physicochemical conditions serve as environmental filters, selecting for microbial communities that are thermotolerant or thermophilic and sustained by CO_2_ fixation, sulfur cycling, and other chemolithotrophic processes [28]. Although numerous studies have reported the microbial diversity and metabolic potential of hot spring communities [29–41], no prior work has systematically examined these communities through a biotechnology lens, despite recent acknowledgement of their potential [28]. Previous metagenomic studies of hot spring microbiomes have largely focused on thermostable enzyme discovery [2, 42–44], regional patterns in community structure and metabolism [31, 35, 45–50], and the influence of physicochemical parameters on microbial diversity [33]. While these studies were not designed to address CO_2_ utilisation, they collectively provie a rich and underexploited resource that can be reanalysed to assess the biotechnological potential of hot spring microbiomes in this context.

To evaluate the biotechnological potential of hot spring microbiomes for CO_2_ valorisation, we reanalysed 72 publicly available and one newly sequenced terrestrial hot spring metagenomes spanning a wide range of temperatures and pH. We evaluated the abundance of genes associated with CO_2_ fixation, energy metabolism, and the biosynthesis of value-added products, enabling us to disentangle how environmental constrains shape both microbial diversity and biotechnological promise. By linking community structure to functional potential, we provide a blueprint for harnessing these microbiomes for future microbiome engineering and CO_2_-utilisation strategies.

## Results and discussion

### Curation of a global terrestrial hot spring metagenomic database

**Fig. 1:**
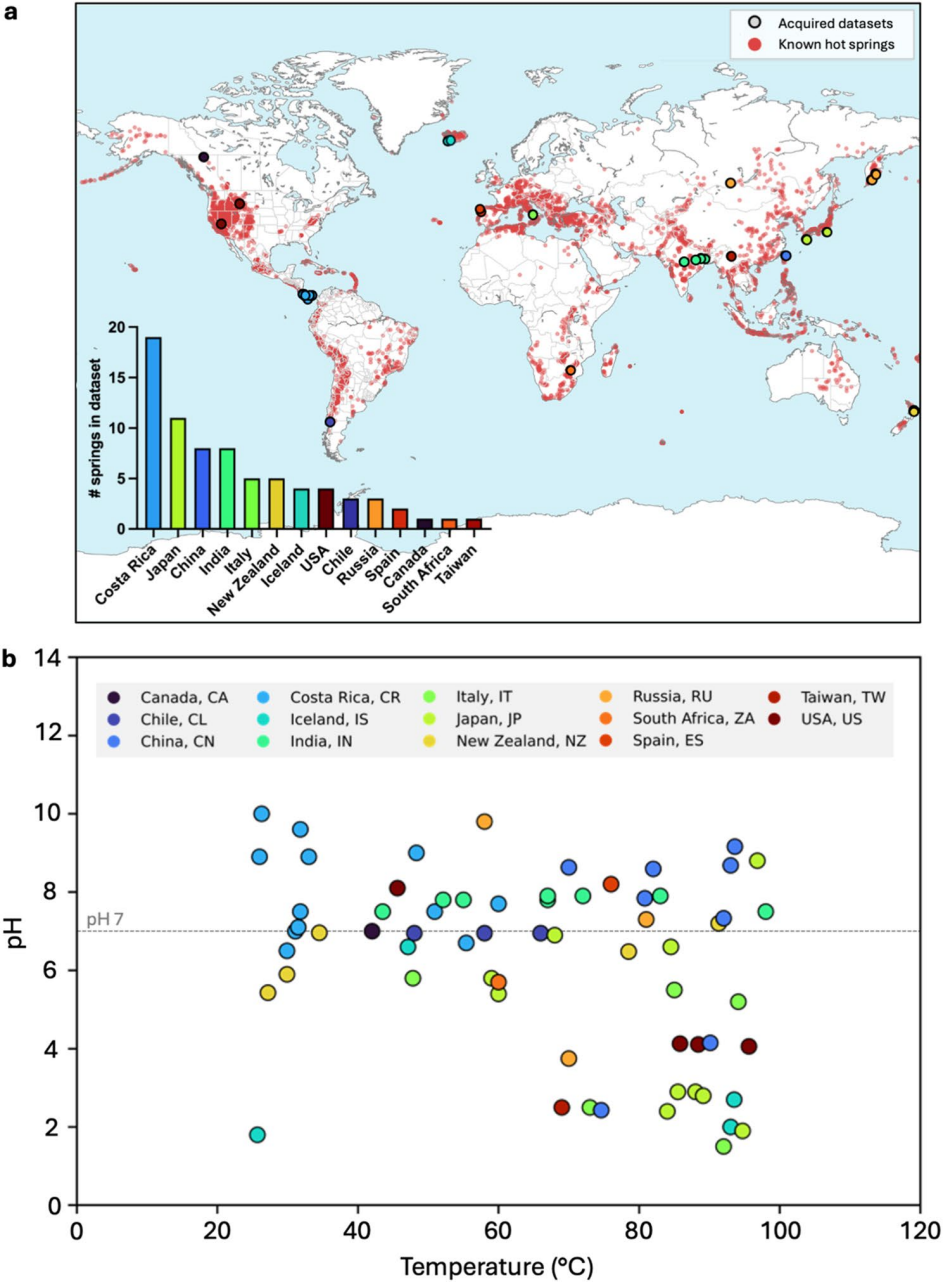
Geographic distribution, pH, and temperature of the 73 hot springs represented in the database. Sequencing datasets were sourced from SRA (except for the newly sequenced Icelandic dataset). **a** Map of hot spring locations. Red translucent circles indicate known hot spring sites (not including Antarctica) [51]; opaque coloured circles represent springs included in this study, colour-coded by country/region. **b** Distribution of pH and temperature for the represented springs. pH 7 is indicated by the grey line

Our curated hot spring database includes 72 metagenomic datasets sourced from public databases (Supplemental File 1) and one newly sequenced dataset, totalling 2.3 Tb in read file size. These datasets span 14 countries and regions, and represent all continents with geothermal activity (Fig. 1a): Canada (‘CA’, *n* = 1), Chile (‘CL’, *n* = 3), China (‘CN’, *n* = 8), Costa Rica (‘CR’, *n* = 18), Iceland (‘IS’, *n* = 4), India (‘IN’, *n* = 8), Italy (‘IT’, *n* = 5), Japan (‘JP’, *n* = 10), New Zealand (‘NZ’, *n* = 5), Russia (‘RU’, *n* = 3), South Africa (‘ZA’, *n* = 1), Spain (‘ES’, *n* = 2), Taiwan (‘TW’, *n* = 1), and the USA (‘US’, *n* = 4). Environmental parameters range from pH 1.5 to 10.0 and temperatures 25.7°C to 98°C, with over half the datasets originating from acidic springs (*n* = 38) (Fig. 1b). Samples are designated by their temperature, pH, and country/region of origin in the format: T_pH_countrycode (e.g., the new Icelandic spring dataset ‘BS1’ is labelled 47.1°C_pH6.6_IS).

Raw read counts per metagenome (sum of forward and reverse) range from 1.2 million to 387.5 million, with mean and median counts of 85.2 and 63.2 million reads, respectively. After trimming, over 90% of reads were retained across most datasets, with an average read retention of 95.3%.

Assembled metagenomes ranged in total length from 4.25 Mb to 325.25 Mb with assembly size decreasing with increasing temperature and metagenomes sequenced from hot springs at or above 73 °C characterised by assembly lengths shorter than 150 Mb.

### Globally distributed hot spring microbiomes exhibit temperature-driven taxonomic and metabolic stratification

**Fig. 2:**
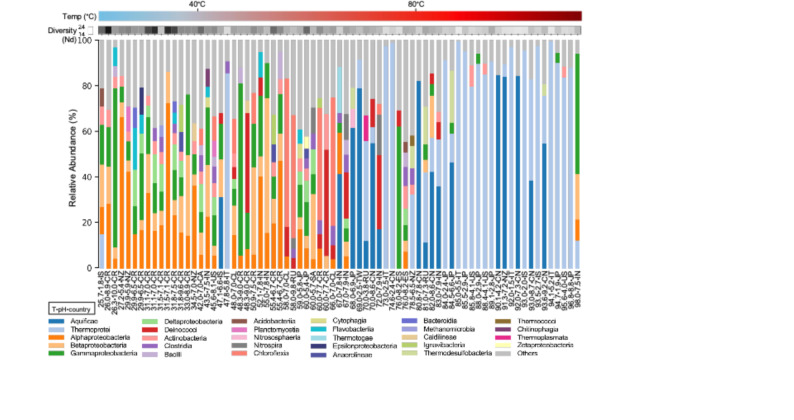
Relative abundance of taxa at the class level in hot springs. Taxa representing < 4% of total reads are grouped as “Others”. Metagenomes are ordered by ascending temperature from left to right. Diversity (Nonpareil index, N_d_) is indicated above each bar. Sample IDs include temperature, pH, and country/region of origin (e.g., 47.1_6.6_IS). Country alpha-2 codes include: Canada, CA; Chile, CL; China, CN; Costa Rica, CR; Iceland, IS; India, IN; Italy, IT; Japan, JP; New Zealand, NZ; Russia, RU; South Africa, ZA; Spain, ES; Taiwan, TW; USA, US

We identified 81 taxa at the class level across the 73 metagenomes. Relative abundance analysis, performed using Kaiju, revealed broad shifts in taxonomic composition from cooler to hotter springs, along with a decline in community diversity (Fig. 2, Supplemental File 2). A more detailed plot with a < 1% taxa filter can be found in Supplemental Figure S3. Proteobacterial classes (Alpha-, Beta-, Gamma-, and Deltaproteobacteria) dominated at temperatures below 67 °C, alongside Actinobacteria, Clostridia, and Bacilli. At higher temperatures, Aquificae and Thermoprotei emerged as dominant taxa, while Proteobacteria (Pseudomonodota) persisted at lower relative abundances. A notable exception to this trend is sample 98.0-7.5-IN (SRR3961740), the highest temperature spring in our dataset, which exhibits an anomalously high abundance of Gammaproteobacteria alongside Thermoprotei. While the latter is expected at this temperature, the presence of Proteobacteria, typically enriched in mesophilic conditions [52], suggests potential contamination or variation in sampling method.

Between communities, principal component analysis (PCA) highlighted the clustering of microbial communities based on temperature and pH (Supplemental Figure S1, Supplemental File 3). Proteobacterial communities clustered at moderate temperatures (~ 47.2 °C) and near-neutral pH (~ 5.8), while Thermoprotei and Aquificae communities were enriched in higher-temperature springs (average 85.9 °C and 84.6 °C), as seen in originating studies [45, 53]. A significant negative correlation between Thermoprotei abundance and pH (*r* = − 0.69, *p* < 0.0001) underscores the adaptation of this archaeal class to acidic conditions. In contrast, Aquificae inhabited a broader pH and temperature range, consistent with its presence across diverse environmental gradients [54–56]. Co-occurrence analysis between taxa provided further evidence for temperature-driven selection (Figure S2), with Thermoprotei exhibiting strong negative correlations with most taxa, aside from other thermophilic archaea, highlighting its specialised adaptation to high-temperature environments. In contrast, Aquificae positively correlates with a wide range of taxa, likely as extension of a broader pH and temperature tolerance. These findings are consistent with previous studies that highlight specialism of Thermoprotei in acidic hyperthermophilic springs (≥ 80 °C) compared to relative generalism by Aquificae inhabiting moderately (45–70 °C) and extreme thermophilic (≥ 70 °C) springs [54–58], though other studies have also described nutrient availability as a factor [33]. Qi et al. [59] provide strong evidence that pH and temperature are the primary factors influencing general bacterial and archaeal abundance, with archaea more abundant at extremes of pH. Additionally, a recent study of Yellowstone hot spring communities examined the effects of 64 geochemical analytes on community composition and confirmed that pH, followed by temperature, are the two most influential factors in broadly shaping microbial community structure [41]. Taken together, these results underscore the fundamental role of temperature and pH as the key environmental drivers of microbial community structure in geographically dispersed hot spring habitats.

From a biotechnological perspective, this suggests that high temperature and low pH conditions of some industrial waste gases may be tolerated better by archaeal communities. The ability of thermophillic archael communities to upgrade raw carbon rich waste gas is achieving success at full scale plant level [60]. An example highlighting the metabolic utility and tolerance of an archael species to carbon rich waste gas was shown by Kim et al. (2016) where the carboxydotrophic archaea *Thermococcus onnurineus* converted toxic steel-mil gas into H_2_ and CO_2_ off gas. Conidtioned gas was then able to be converted to acetic acid by bacterial *Thermoanaerobater kivui* in a two stage bioreactor [61].

For greater predictive strength, co-occurrence was assessed within PCA clusters. Generalisable taxonomic structures across these communities were observed for example Aquificae dominated cluster, Aquificae most positively correlated with Gammaproteobacteria (*r* = 0.90, *p* = < 0.05), Cytophagia (*r* = 0.90, *p* = < 0.05), and Hydrogenophilalia (*r* = 0.90, *p* = < 0.05), Supplemental File 4. Comparatively the Thermoprotei cluster Thermoprotei are are not statistically significantly, positively correlated with any other classes showing their isolated lifestyle. Co-occurrence of taxa may indiciate cross feeding cross feeding wich can give rise to division of labour and robustness in *ex-situ* growth of communities, a beneficial trait for biotechnological applications. Further to this understanding, the effect of the selective pressure of temperature and pH to choose different communities can be used to preferentially obtain or enrich communities of interest with specific metabolic functions.

Beyond the enrichment of distinct microbial groups, a broader trend of decreasing diversity was observed along the global temperature gradient. Higher temperatures were significantly associated with lower community diversity, as indicated by the Nonpareil index (*N*_*d*_, *r* = − 0.56, *p* = 2.91 × 10^–14^) (Fig. 2). This global inverse relationship confirms multiple previous regional studies of terrestrial hot springs [54, 62–68].

Overall, our analyses highlights the trends in community structure in relation to physicochemical niches. A significant decline in community diversity with increasing temperature at the globally distributed hot springs was observed. The analysis serves as a resource for identifying potential microbial communities with tolerances to CO_2_ rich waste gas.

### High temperatures drive specialisation in microbial metabolism

**Fig. 3:**
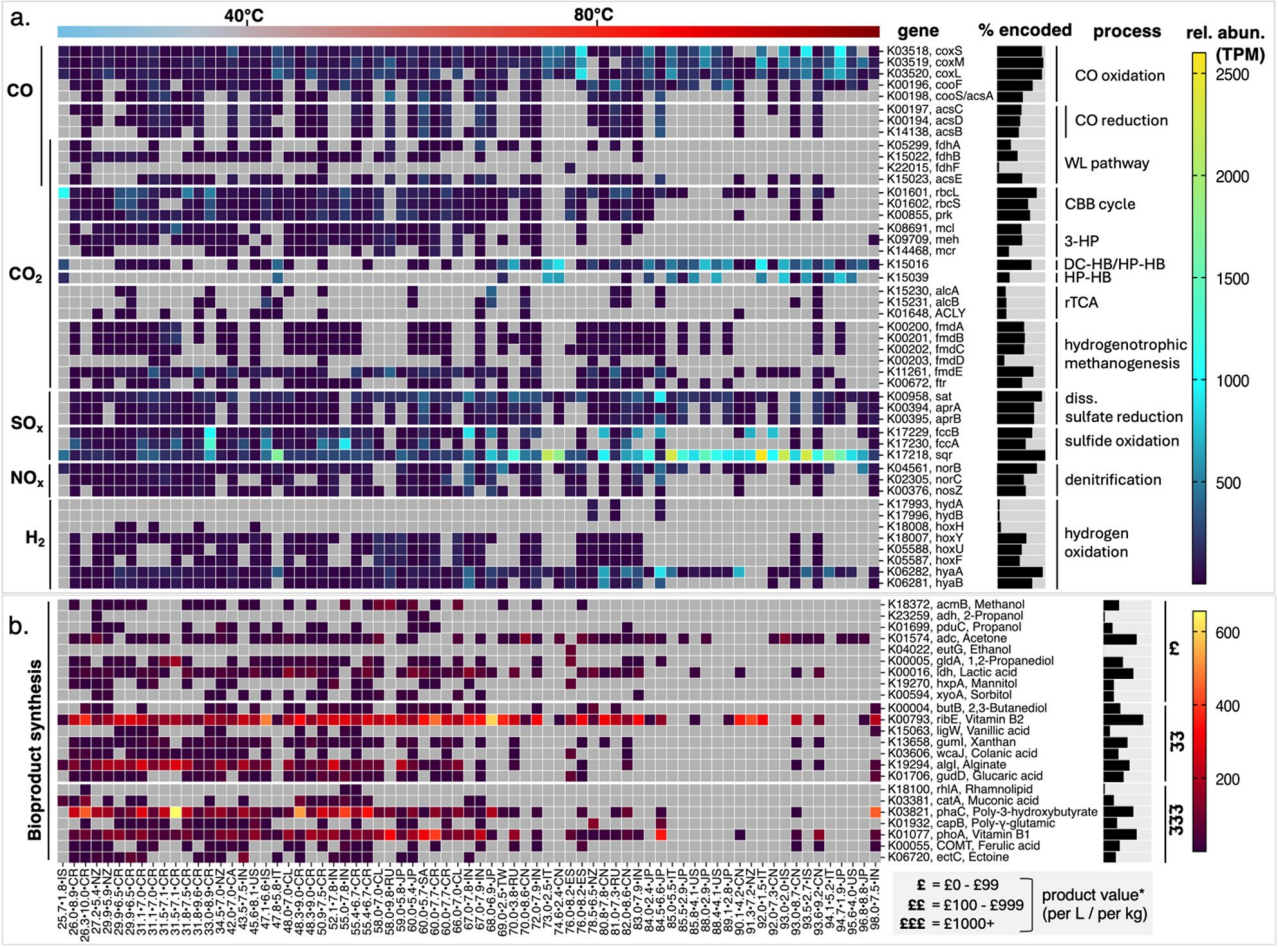
Heatmap of metagenomic mapped gene abundance (expressed as Transcripts per Million, TPM) of genes encoding enzymes implicated in the utilisation of industrial waste gases (**a**) and biosynthesis of value-added products (**b**), including indicative product values. *Value brackets are based on product prices listed on Merck in September 2024. Percent encoded black bars to the right of each gene indicate the proportion of samples (out of 73) that genes were detected in. Metagenomes are ordered by ascending temperature. Gene abundance values of 0 (not detected) are coloured grey. Sample IDs include temperature, pH, and country/region of origin (e.g., 47.1_6.6_IS). * Country alpha-2 codes include: Canada, CA; Chile, CL; China, CN; Costa Rica, CR; Iceland, IS; India, IN; Italy, IT; Japan, JP; New Zealand, NZ; Russia, RU; South Africa, ZA; Spain, ES; Taiwan, TW; USA, US

To assess the metabolic potential of hot spring microbiomes regardless of the effect on diversity, we surveyed the presence and abundance of genes encoding key metabolic processes of relevance to developing new CO_2_–rich waste gas valorisation biotechnologies using whole communities. Community level views are advantageous as they incorporate cross feeding potential, commonly autotroph-heterotroph, and give an estimation of products that can be synthesised as a community that single strains cannot. The analysis focuses on the genomic potential to use industrial waste gases (CO, CO_2_, H_2_, NOx, SOx), and the potential to generate bioproducts of added value (Fig. 3, Supplemental File 5).

Across the surveyed metagenomes, we assessed the metagenomic mapped gene abundance of genes encoding key carbon fixation pathways, including the Wood-Ljungdahl (WL) pathway, reductive TCA (rTCA) cycle, 3-hydroxypropionate (3-HP) bi-cycle, 4-hydroxybutyrate/3-hydroxypropionate (HB-HP), dicarboxylate/4-hydroxybutyrate (DC-HB) cycle and the Calvin-Benson-Bassham (CBB) cycle (see Supplemental File 6 for full pathway TPM data per sample). We also assessed hydrogenotrophic methanogenesis alongside these, given the potential for organisms with this capability to valorise CO_2_ into methane. Our findings show a clear shift from diverse carbon fixation pathways at lower temperatures (e.g., CBB cycle in mesophilic and lower thermophilic ranges, consistent to originating study [45]) to more specialised pathways like the WL pathway at higher temperatures. Genes encoding key enzymes in CO oxidation, nitrogen and sulfur cycling (e.g. *sqr* implicated in sulfide oxidation) are more abundant in high-temperature hot spring microbiomes. This is concomitant with the taxonomic shift to Aquificae, Thermoprotei and a reduction in diversity, indicating metabolic specialisation by these archaeal-dominated lower diversity microbiomes (Figs. 2 and 3).

Annotation of metagenome-assembled genomes (MAGs) corroborated these findings (Supplemental File 8). Of the 44 Aquificae MAGs 10 representatives encoded genes for CO_2_ fixation via the rTCA pathway and 38 encoded *sqr*. Comparitevely 194 Thermoprotei MAGs were identified, 145 showed evidence of CO oxidation, 106 for genomic evidence of CO_2_ fixation via the DC-HB/HP-HB cycle, and 127 dissimilatory sulfate reduction/sulfide oxidation. Energy yielding hydrogen oxidation, highly amenable to biotechnological applications, was present in Aquificae [47] and Thermoprotei MAGs .Strong evidence for MAGs possessing mesophilic metabolism, such as the CBB cycle, was seen in Cyanobacteriia, and Deinococci, Alphaproteobacteria, and Gammaproteobacterial.

Despite this clear trend, some genes and pathways are broadly distributed across the temperature scale. For instance, CO oxidation, sulfide oxidation and the reduction of sulfate appear to be universal capabilities encoded in the hot spring metagenomes surveyed. Similarly, the key gene *acsB* in the WL pathway, and K15016 in the dicarboxylate/4-hydroxybutyrate (DC-HB) and 4-hydroxybutyrate/hydroxypropionate (HP-HB) pathways, are abundant throughout (Fig. 3).

Overall, key capabilities such as CO_2_, CO sequestration along with NOx and SOx metabolism are common in hot spring microbiomes regardless of temperature, highlighting the applicability of these communities for CO_2_ sequestration through microbial biotechnology approaches.

### Hot spring microbiomes are untapped resources for bioproduct synthesis

For successful microbiome-based CO_2_ valorisation, the community must possess the relevant metabolic capabilities to upgrade CO_2_ to useful bioproducts. Communities with a diverse product range allow for a more flexible scope for engineering, or a multi-product fermentation, though ultimately only one product is needed for successful implementation. The surveyed metagenomes reveal significant genomic potential for the biosynthesis of value-added products, with relative abundances varying across the temperature gradient (Fig. 3b). Among these, the most prevalent biosynthesis gene is *ribE*, associated with vitamin B2 production, detected in 61 of the 73 metagenomes and found at consistently high abundances across the temperature spectrum. The *ribE* gene was detected in the majority of bacterial MAGs (Supplemental File 8) whereas it was present in no archaeal MAGs aside from those of the Nitrososphaerales order. Similarly widespread is *adc*, a gene linked to acetone production, identified in 50 of the 73 metagenomes and encoded by MAGs identified as Thermoprotei. Acetone is a versatile, industrially significant chemical used as a solvent and a precursor in the production of plastics, pharmaceuticals and synthetic fibres [69, 70]. It is already recognised as a biotechnologically derived value-added product through acetogen pathways [13]. Further to this, the metabolism is consistently supported in hyperthermophilic (≥ 80 °C) acid conditions, making it applicable for waste gas applications.

Other potential value-added products include lactic acid (*ldh*), an important precursor chemical for a wide range of industrial applications; vitamin B1 (*phoAB*), used in dietary supplements and food fortification; and poly-3-hydroxybutyrate (*phaC*), a key building block for biodegradable plastics, medical implants and cosmetics [71]. The *phaC* gene was highly abundant in the XXX Alphaproteobacterial and Gammaproteobacterial classes. Also encoded at relatively high abundances is *algI*, implicated in alginate synthesis. Alginate is used as a thickening, emulsifying, and gel-forming agent in several industrial applications [72]. Interestingly, few publications discuss the presence of alginate in hot springs [73] though *algI* has a high relative abundance in most springs below 66 °C. This gene was not detected in Aquificae or Thermoprotei MAGs but present abundant in many other classes including Bacilli, Clostridia, Bacteroidia, Ignavibacteria, Anaerolineae, Cyanobacteriia, Plantomycetia, Alphaproteobacteria, Gammaproteobacteria, Kiritimatiellia, and Verrucomicrobiae.

**Fig. 4:**
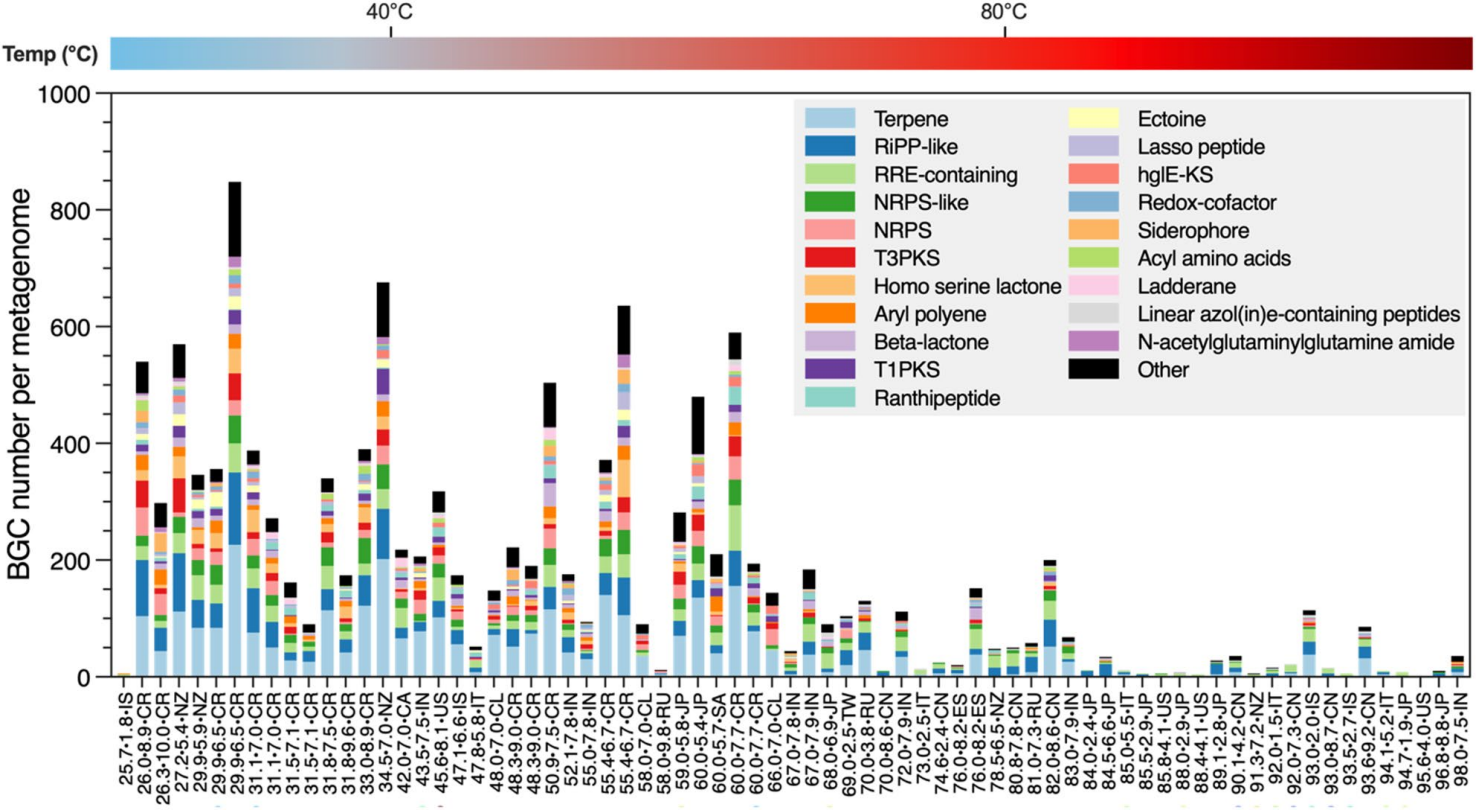
Number and type of biosynthetic gene clusters (BGC) present in each metagenome ranked in ascending order based on temperature. Metagenome IDs are composed of the temperature of the spring, pH, and the country/region of origin. Country alpha-2 codes include: Canada, CA; Chile, CL; China, CN; Costa Rica, CR; Iceland, IS; India, IN; Italy, IT; Japan, JP; New Zealand, NZ; Russia, RU; South Africa, ZA; Spain, ES; Taiwan, TW; USA, US. *RiPP* ribosomally-synthesised and post-translationally modified peptides, *RRE* RiPP recognition element, *NRPS* non-ribosomal peptide synthetase, *T3PKS* type III polyketide synthetase, *T1PKS* type I polyketide synthetase, *hgle-KS* heterocyst glycolipid synthetase. Full details of identified BGCs can be found in Supplemental File 7

Microorganisms produce specialised (secondary) metabolites through the expression of genes on biosynthetic gene clusters (BGCs). Resulting compounds are not required for growth, but provide ecological advantages, serving such roles as antimicrobial defence [74], iron acquisition (siderophores; [75]), population control (bacteriocins; [76]), cell signalling (quorum sensing; [77]), and stress resistance (e.g., ectoine; [78]). Many of these bioactive compounds have high societal value, with applications in pharmaceuticals, agriculture, food, cosmetics, bioremediation and biofuels. We explored the potential of hot spring microbiomes to produce such high value compounds by analysing the distribution and abundance of BGCs using antiSMASH (Fig. 4).

Across the dataset, a total of 567 unique secondary metabolites were identified. Overall, bioproduct metabolic richness declined with increasing temperature. The number of BGCs per metagenome ranged from 848 in sample 29.9°C_pH5.9_NZ to just 2 in sample 95.6°C_pH4.06_US. Almost all springs have the metabolic potential to generate at least one single value-added compound. Cooler springs may benefit from potentially greater choice or multi product fermentations, though the trade off to this is that cooling would be required for carbon rich waste gas.

Terpenes were the most abundant BGC type, with a maximum 226 clusters in sample 29.9°C_pH5.9_NZ. Terpenes are a highly versatile group of hydrocarbons that have roles in cell signalling, competition and defence, stress response, and host interaction [79]. They can also serve as a wide range of valuable properties, including anti-inflammatory, anticancer, antibiotic and antioxidant properties [80–83]. Some terpenes serve as common flavouring agents (e.g. menthol) and colourants in food and beverages, while others are used in pest control and biofuel production [80, 81, 84]. Their abundance in these hot spring communities demonstrates significant potential to harness terpene bioproduction for wide ranging applications in multiple industrial settings, and further research should address the range and value of individual terpenes these BGCs encode.

The most common BGC-encoding single product identified is ectoine (716 annotations across 17 samples; Fig. 4), a high value osmolyte implicated with protection against oxidative stress, high salinities and thermal extremes [85, 86] and is widely used in cosmetics and anti-inflammatory drugs [87]. Non-ribosomal peptides were also abundant, producing antimicrobials (e.g., icosalide, derived from bacterial symbionts; [88]), siderophores (e.g., crochelin), antifungals (e.g., fengycin; [89]), and anticancer drugs (e.g., vioprolide A and microsclerodermin; [90, 91]). Notably, communities from the most extreme environments still exhibited potential for valuable bioproduct biosynthesis. For instance, sample 93.0°C_pH2.0_IS – presumably representing a community of hyperthermophilic acidophiles – encoded BGCs for anticancer cyclolipodepsipeptides [90], fungal signalling molecule ralsolamycin [92], and the lipocyclopeptide antibiotic icosalide [88].

Quantification of secondary metabolism of hot springs at the microbiome level remains scarce [40, 93], with some studies focusing on specialsed members, such as cyanobacteria [46]. To our knowledge, this is the first study to quantify secondary metabolism of hot spring microbiomes at a global scale. Consistent with primary metabolism, we observed a decline in metabolic richness with increasing temperature. Similar trends have been noted regionally, such as in the study by Das et al. (2018), which attributed the decline in part to the instability of antibiotics at higher temperatures. Ruhl et al. (2019) further demonstrated, using 16 S rRNA gene sequencing and Pfam-based analyses, that increasing temperature correlates with reduced species and metabolic diversity, likely due to the selection of thermostable enzymes and fewer species with smaller genomes [94, 95]. Similarly, in cyanobacteria BGCs represented a smaller proportion of the genome in hot spring environments as compared to non-thermal environments [46].

Despite the reduced diversity of bioproducts in high temperature springs genome mining efforts, using tools like antiSMASH [96], have identified BGCs in extremophiles, encoding compounds such as antibiotics, anticancer agents and antifungals [97]. Others have identified novel compounds, such as hectochlorin from cyanobacteria [98] and a lipophilic cyclopeptide antibiotic produced by *Thermoactinomyces vulgaris*, isolated from an Icelandic hot spring [99]. Modern meta-omics approaches have further expanded the discovery of BGCs in unculturable microorganisms, revealing extensive biosynthetic pathways linked to ecological adaptation in hot springs [100, 101].

Our results corroborate these observations and demonstrate that globally distributed hot spring microbiomes harbour the capability to produce high-value secondary metabolites, with greater richness observed in springs below 70 °C. This biotechnological potential underscores the value of hot spring microbiomes as platforms for industrial applications. Future work to culture stable communities on simulated waste gases and validate the biosynthesis of bioproducts is a key next step on the path towards a viable industrial scale CO_2_ valorisation technology.

## Conclusions

Our global study has highlighted the universal potential of hot spring microbiome communities for *ex-situ* bioreactor based valorisation of CO_2_-rich waste streams. Deployment of biotechnologies stemming from these communities could reduce demand for energy-intensive cooling (the largest cost to bioprocessing) and create value from waste gases generated by sectors like cement and steel production [102]. Metagenomic and genomic evidence describe a wide spectrum of microbial communities within hot springs. Less extreme environments harbour metabolically rich communities, capable of CO_2_ sequestration at lower temperatures, offer high diversity, potentially multi product valorisaiton approaches. Extreme communities show evidence for being highly adapted to detoxify and/or sequester gas impurities SO_x_, NO_x_, H_2_S and CO (Fig. 3a) while tolerating waste gas like conditions, extreme pH and temperature, especially archaea-dominated communities.

## Materials and methods

### Sampling and analysis of Icelandic spring microbial mats

In addition to 72 datasets recovered from the Sequence Read Archive (SRA; detailed below), we included a newly sequenced dataset deriving from samples we collected and processed (‘BS1’). This metagenomic dataset was generated from DNA extracted from water and biomass from a spring in Ölkelduháls, Iceland. pH and temperature of the site were measured using handheld probes and were found to be 47.1 °C and pH 6.6, respectively. Biomass samples (microbial mats and streamers) were collected in September 2021 using sterile tweezers into sterile 50 mL Falcon Tubes and immediately frozen. DNA was extracted using the Qiagen DNeasy PowerBiofilm Kit at the St Andrews University, following manufacturer’s instructions. DNA was quantified using the Qubit dsDNA High Sensitivity (HS) Assay Kit (Life Technologies). Extraction yielded 9.98 ng/uL. The library was prepared from 50 ng DNA using Illumina DNA Prep, (M) Tagmentation library preparation kit (Illumina) following the manufacturer’s instructions. The sample underwent the simultaneous fragmentation and addition of adapter sequences. Following library preparation, the final library concentration was measured using the Qubit dsDNA HS Assay Kit, and was determined to be 19.2 ng/uL. Average library size was determined using the Agilent 2100 Bioanalyser (Agilent Technologies). The library was pooled with 4 others (not included in this study) in equimolar ratios of 0.6nM, and sequenced paired end for 500 cycles using the Illumina NovaSeq 6000, generating 2 × 150 bp reads.

### Acquisition of public data

All other Illumina sequence read files were downloaded from the Sequence Read Archive (SRA) using the SRA Toolkit in December 2022. Samples were selected based on the availability of key metadata such as temperature and pH. In the case of replicated samples, we chose the largest single metagenome to include in our survey. Metagenomes recovered from enrichments initiated with spring communities were considered outside of the scope of the study and not included. Samples, metadata, and associated publications are detailed in Supplementary File 1. While care was taken to be comprehensive in our data collection, we acknowledge that some relevant datasets may have been overlooked. We also note that new metagenomes have been deposited that were not available to us at the time of our analyses. A notable example is the recent study by Colman et al. (2024) that contributes multiple new hot spring metagenomes from Yellowstone National Park to the public databases that we were unable to include in our study [41].

Reanalysis of published data enables us to normalise for any biases that would otherwise be introduced using different bioinformatics tools and parameters therein. Numerous factors relating to sample acquisition, biomass concentration, DNA extraction, library preparation and the size of the library pool can impact on the downstream bioinformatics results; none of which we were involved in (except for BS1 collected and sequenced by us), nor is there metadata associated with these datasets to characterise these differences. Our underlying assumption in the sections that follow is that all datasets following our reanalysis are comparable, though we acknowledge there are limitations in this approach.

### Read trimming, taxonomic classification and metagenomic assembly

Raw sequencing data was trimmed with Trimmomatic, v0.39 [103], with options and thresholds of: ILLUMINACLIP: TruSeq3-PE.fa:2:30:10, LEADING:30, TRAILING:30, SLIDINGWINDOW:4:15, and MINLEN:36. Resulting paired trimmed sequencing reads were taxonomically classified using Kaiju v1.7.2 run in “greedy” mode [104], using taxon identifiers v2019-06-25, and Refseq database v2019-06-25. These include Archaea, Bacteria, and viruses. The proportion of unclassified reads per sample can be found in Supplemental File 2. Trimmed reads were assembled using Spades v3.15.5 [105] using the flag --meta and subsequently filtered to retain contigs larger than 2.5 kb. Trimmed reads were mapped to the assembly using Bowtie2 v2.4.5 [106], then sorted and converted from a sequence alignment map to a sorted binary alignment map using Samtools, v1.16.1 [107]. Duplicates in the binary alignment map were located and removed using Picard v2.27.5 (https://broadinstitute.g.ithub.io/picard/).

### Recovery and analysis of metagenome assembled genomes (MAGs)

Contigs were binned into MAGs using MetaWRAP v1.3.0 [108]. The binning module was used, followed by the refinement module, and the completeion and contamination thresholds were set to 70% and 10% respectively. Refined mags that met the filtering criteria were taxonomically identified using GTDB-Tk v2.4.1 [109] with GTDB release version R220. Coding sequences on the MAGs were identified using Prokka v1.14.6 [110], and annotated using KofamScan v1.3.0 [111].

### Estimation of microbial diversity

Microbial diversity was quantified from metagenomes using the Nonpareil index of sequence diversity (*N*_*d*_) [112, 113]. Nonpareil v3.4.1 was used with the -T alignment option to obtain the redundancy summary file. The *Nd* was calculated from the file using the Nonpareil R package.

### Gene annotation and quantification

Prokka v1.14.6 [110] was used to identify and annotate coding sequences, which were subsequently annotated using Kofamscan v1.3.0 [111]. The general feature format (.gff) files from Prokka were converted to general transfer format (.gtf), and the binary alignment map and general transfer files were used to calculate the count of reads over the gene features in the metagenome using HTSeq v2.0.2 [114]. Read length and gene length were then calculated, and gene abundance was calculated as outlined below. These annotations were merged with the gene abundance calculations to give a final output of gene abundance per sample.

Metagenomic-mapped gene abundance values were calculated using a transcript per million (TPM) caluclation, where *G* is the set of all genes, *r*_*g*_ is reads mapped to gene, *r*_*l*_ is read length, and fl._*g*_ is feature length:

$$T{\text{ }}=~\sum\nolimits_{{g \in G}} {\frac{{{r_g} \times ~rl~}}{{f{l_g}}}} \, \cdot \,TPM=\,\frac{{{r_g} \times ~rl~ \times ~{{10}^{6~}}~}}{{f{l_g} \times ~T}}$$

TPM normalises for both read depth and gene length, enabling comparability across datasets to yield a robust assessment of gene abundance for the metabolic genes of interest [115, 116].

### Identification of biosynthetic gene clusters

Biosynthetic gene clusters (BGCs) were identified using antiSMASH v6.1.1 [97, 117]. The flag --cb-knownclusters was used to identify compounds from known clusters against the MIBiG database. Prodigal [118] was used as the gene-finding tool. Metrics of the assembly, used to contextualise secondary metabolism, were obtained with MetaQUAST using QUAST v5.2.0 [119]. Most BGCs were identified at the strict detection level, with some (“-like” or “-containing” clusters) detected at the relaxed level (details in Supplementary File 2).

### Statistics and data visualization

Linear regression was calculated using linregress from the scipy.stats python library [120]. The principal component analysis and biplot were generated using the sklearn.decomposition module from scikit-learn [121]. The geographic locations of the geothermal springs were visualised using the mpl_toolkits.basemap module from Basemap [122]. General data visualisation and manipulation were performed using Matplotlib [122], Seaborn [123] and Pandas [124].

## Supplementary Information

Below is the link to the electronic supplementary material.

Supplementary Material 1.

Supplementary Material 2. Contains accession numbers and metadata associated with metagenomic datasets used in our meta-analyses.

Supplementary Material 3. Summarises the proportion of reads unclassified by Kaiju for each sample.

Supplementary Material 4. Contains results from PCA analysis of the correlation of class distribution with pH and Temperature.

Supplementary Material 5. Contains Spearman’s Rank of taxa.

Supplementary Material 6. Gives details of the marker genes included in Figure 3.

Supplementary Material 7. Contains metagenomic mapped gene abundance TPM data of all genes in the major carbon fixation pathways discussed in this work.

Supplementary Material 8. Gives details of the Biosynthetic Gene Clusters identified in antiSMASH analysis.

Supplementary Material 9.

## Data Availability

Metagenomic sequencing reads generated for this study have been deposited at the Sequence Read Archive in BioProject PRJNA1082630 with the accession number SRR31758307. Links to publicly archived datasets included in this study are detailed in Supplementary File 1.

## References

[1] Boyd ES, Leavitt WD, Geesey GG. CO(2) uptake and fixation by a thermoacidophilic microbial community attached to precipitated sulfur in a geothermal spring. Appl Environ Microbiol. 2009;75(13):4289–96. 10.1128/AEM.02751-08. 19429558 10.1128/AEM.02751-08 PMC2704841

[2] Paul R, Rogers TJ, Fullerton KM, Selci M, Cascone M, Stokes MH, et al. Complex organic matter degradation by secondary consumers in chemolithoautotrophy-based subsurface geothermal ecosystems. PLoS ONE. 2023;18(8):e0281277. 10.1371/journal.pone.0281277. 37594978 10.1371/journal.pone.0281277 PMC10437873

[3] Collis J, Strunge T, Steubing B, Zimmermann A, Schomäcker R. Deriving economic potential and GHG emissions of steel mill gas for chemical industry. Front Energy Res. 2021;9:642162. 10.3389/fenrg.2021.642162.

[4] Schakel W, Hung CR, Tokheim L-A, Strømman AH, Worrell E, Ramírez A. Impact of fuel selection on the environmental performance of post-combustion calcium looping applied to a cement plant. Appl Energy. 2018;210:75–87. 10.1016/j.apenergy.2017.10.123.

[5] Więcław-Solny L, Tatarczuk A, Stec M, Krótki A. Advanced CO2 capture pilot plant at Tauron’s coal-fired power plant: initial results and further opportunities. Energy Procedia. 2014;63:6318–22. 10.1016/j.egypro.2014.11.664.

[6] Seyed Hosseini N, Shang H, Scott JA. Biosequestration of industrial off-gas CO2 for enhanced lipid productivity in open microalgae cultivation systems. Renew Sustain Energy Rev. 2018;92:458–69. 10.1016/j.rser.2018.04.086.

[7] Aslam A, Thomas-Hall SR, Manzoor M, Jabeen F, Iqbal M, Uz Zaman Q, et al. Mixed microalgae consortia growth under higher concentration of CO(2) from unfiltered coal fired flue gas: Fatty acid profiling and biodiesel production. J Photochem Photobiol B. 2018;179:126–33. 10.1016/j.jphotobiol.2018.01.003. 29367147 10.1016/j.jphotobiol.2018.01.003

[8] Kikas T, Olt J, Podkuiko L. Growth of Scenedesmus obliquus under artificial flue gas with a high sulphur concentration neutralized with oil shale ash. Proc Est Acad Sci. 2017;66(2):151-8; 10.3176/proc.2017.2.03

[9] Duarte JH, Fanka LS, Costa JAV. Utilization of simulated flue gas containing CO2, SO2, NO and ash for Chlorella fusca cultivation. Bioresour Technol. 2016;214:159–65. 10.1016/j.biortech.2016.04.078. 27132223 10.1016/j.biortech.2016.04.078

[10] Kao CY, Chen TY, Chang YB, Chiu TW, Lin HY, Chen CD, et al. Utilization of carbon dioxide in industrial flue gases for the cultivation of microalga Chlorella sp. Bioresour Technol. 2014;166:485–93. 10.1016/j.biortech.2014.05.094. 24950094 10.1016/j.biortech.2014.05.094

[11] Lee J-S, Kim D-K, Lee J-P, Park S-C, Koh J-H, Cho H-S, et al. Effects of SO2 and NO on growth of Chlorella sp. KR-1. Bioresour Technol. 2002;82(1):1–4. 10.1016/s0960-8524(01)00158-4. 11848373 10.1016/s0960-8524(01)00158-4

[12] Liang Y, Tang J, Luo Y, Kaczmarek MB, Li X, Daroch M. Thermosynechococcus as a thermophilic photosynthetic microbial cell factory for CO(2) utilisation. Bioresour Technol. 2019;278:255–65. 10.1016/j.biortech.2019.01.089. 30708328 10.1016/j.biortech.2019.01.089

[13] Liew F, Martin ME, Tappel RC, Heijstra BD, Mihalcea C, Kopke M. Gas fermentation- a flexible platform for commercial scale production of low-carbon-fuels and chemicals from waste and renewable feedstocks. Front Microbiol. 2016;7:694. 10.3389/fmicb.2016.00694. 27242719 10.3389/fmicb.2016.00694 PMC4862988

[14] Moreira JPC, Diender M, Arantes AL, Boeren S, Stams AJM, Alves MM, et al. Propionate production from carbon monoxide by synthetic cocultures of Acetobacterium wieringae and propionigenic Bacteria. Appl Environ Microbiol. 2021;87(14):e0283920. 10.1128/AEM.02839-20. 33990298 10.1128/AEM.02839-20 PMC8231444

[15] Andreides D, Lopez Marin MA, Zabranska J. Selective syngas fermentation to acetate under acidic and psychrophilic conditions using mixed anaerobic culture. Bioresour Technol. 2024;394:130235. 10.1016/j.biortech.2023.130235. 38141884 10.1016/j.biortech.2023.130235

[16] Rafieenia R, Atkinson E, Ledesma-Amaro R. Division of labor for substrate utilization in natural and synthetic microbial communities. Curr Opin Biotechnol. 2022;75:102706. 10.1016/j.copbio.2022.102706. 35255422 10.1016/j.copbio.2022.102706

[17] Roell GW, Zha J, Carr RR, Koffas MA, Fong SS, Tang YJ. Engineering microbial consortia by division of labor. Microb Cell Fact. 2019;18(1):35. 10.1186/s12934-019-1083-3. 30736778 10.1186/s12934-019-1083-3 PMC6368712

[18] Che S, Men Y. Synthetic microbial consortia for biosynthesis and biodegradation: promises and challenges. J Ind Microbiol Biotechnol. 2019;46(9–10):1343–58. 10.1007/s10295-019-02211-4. 31278525 10.1007/s10295-019-02211-4

[19] Bhatia SK, Bhatia RK, Choi YK, Kan E, Kim YG, Yang YH. Biotechnological potential of microbial consortia and future perspectives. Crit Rev Biotechnol. 2018;38(8):1209–29. 10.1080/07388551.2018.1471445. 29764204 10.1080/07388551.2018.1471445

[20] Levy-Booth DJ, Navas LE, Fetherolf MM, Liu LY, Dalhuisen T, Renneckar S, et al. Discovery of lignin-transforming bacteria and enzymes in thermophilic environments using stable isotope probing. ISME J. 2022;16(8):1944–56. 10.1038/s41396-022-01241-8. 35501417 10.1038/s41396-022-01241-8 PMC9296663

[21] Escuder-Rodríguez JJ, DeCastro ME, Saavedra-Bouza A, González-Siso MI, Becerra M. Bioprospecting for Thermozymes and Characterization of a Novel Lipolytic Thermozyme Belonging to the SGNH/GDSL Family of Hydrolases. Int J Mol Sci. 2022;23(10). 10.3390/ijms23105733. 10.3390/ijms23105733 PMC9145741 35628544

[22] Deive FJ, Alvarez MS, Sanromán MA, Longo MA. North Western Spain hot springs are a source of lipolytic enzyme-producing thermophilic microorganisms. Bioprocess Biosyst Eng. 2013;36(2):239–50. 10.1007/s00449-012-0780-7. 22763779 10.1007/s00449-012-0780-7

[23] Ortega-Villar R, Escalante A, Astudillo-Melgar F, Lizarraga-Mendiola L, Vazquez-Rodriguez GA, Hidalgo-Lara ME, et al. Isolation and Characterization of Thermophilic Bacteria from a Hot Spring in the State of Hidalgo, Mexico, and Geochemical Analysis of the Thermal Water. Microorganisms. 2024;12(6). 10.3390/microorganisms12061066. 10.3390/microorganisms12061066 PMC11205571 38930448

[24] Valdez-Nuñez LF, Rivera-Jacinto MA. Thermophilic bacteria from Peruvian hot springs with high potential application in environmental biotechnology. Environ Technol. 2022;1–16. 10.1080/09593330.2022.2143293. 10.1080/09593330.2022.2143293 36356186

[25] Sarkar A, Chatterjee A, Mandal S, Chattopadhyay B. An alkaliphilic bacterium BKH4 of Bakreshwar hot spring pertinent to bioconcrete technology. J Appl Microbiol. 2019;126(6):1742–50. 10.1111/jam.14236. 30817048 10.1111/jam.14236

[26] Mohammad BT, Al Daghistani HI, Jaouani A, Abdel-Latif S, Kennes C. Isolation and Characterization of Thermophilic Bacteria from Jordanian Hot Springs: Bacillus licheniformis and Thermomonas hydrothermalis Isolates as Potential Producers of Thermostable Enzymes. Int J Microbiol. 2017;2017:6943952. 10.1155/2017/6943952. 29163641 10.1155/2017/6943952 PMC5661075

[27] Burton MR, Sawyer GM, Granieri D. Deep Carbon Emissions from Volcanoes. Rev Mineral Geochem. 2013;75(1):323–54. 10.2138/rmg.2013.75.11.

[28] Galani A, Sipkema D, Sousa DZ. Hot prospects: harnessing thermophilic microbes for syngas fermentation. Trends Biotechnol. 2025. 10.1016/j.tibtech.2025.04.017. 40425413 10.1016/j.tibtech.2025.04.017

[29] Takacs-Vesbach C, Inskeep WP, Jay ZJ, Herrgard MJ, Rusch DB, Tringe SG, et al. Metagenome sequence analysis of filamentous microbial communities obtained from geochemically distinct geothermal channels reveals specialization of three aquificales lineages. Front Microbiol. 2013;4:84. 10.3389/fmicb.2013.00084. 23755042 10.3389/fmicb.2013.00084 PMC3665934

[30] Paul S, Cortez Y, Vera N, Villena GK, Gutiérrez-Correa M. Metagenomic analysis of microbial community of an Amazonian geothermal spring in Peru. Genomics Data. 2016;9:63–6. 10.1016/j.gdata.2016.06.013. 27408814 10.1016/j.gdata.2016.06.013 PMC4932623

[31] Saxena R, Dhakan DB, Mittal P, Waiker P, Chowdhury A, Ghatak A, et al. Metagenomic analysis of hot springs in central India reveals hydrocarbon degrading thermophiles and pathways essential for survival in extreme environments. Front Microbiol. 2016;7:2123. 10.3389/fmicb.2016.02123. 28105025 10.3389/fmicb.2016.02123 PMC5214690

[32] Fortney NW, He S, Converse BJ, Boyd ES, Roden EE. Investigating the Composition and Metabolic Potential of Microbial Communities in Chocolate Pots Hot Springs. Front Microbiol. 2018;9. 10.3389/fmicb.2018.02075. 10.3389/fmicb.2018.02075 PMC6137239 30245673

[33] Nishiyama E, Higashi K, Mori H, Suda K, Nakamura H, Omori S, et al. The relationship between microbial community structures and environmental parameters revealed by metagenomic analysis of hot spring water in the Kirishima area, Japan. Front Bioeng Biotechnol. 2018;6:202. 10.3389/fbioe.2018.00202. 30619848 10.3389/fbioe.2018.00202 PMC6306410

[34] Pedron R, Esposito A, Bianconi I, Pasolli E, Tett A, Asnicar F, et al. Genomic and metagenomic insights into the microbial community of a thermal spring. Microbiome. 2019;7(1):8. 10.1186/s40168-019-0625-6. 30674352 10.1186/s40168-019-0625-6 PMC6343286

[35] Iacono R, Cobucci-Ponzano B, De Lise F, Curci N, Maurelli L, Moracci M, et al. Spatial metagenomics of three geothermal sites in pisciarelli hot spring focusing on the biochemical resources of the microbial consortia. Molecules. 2020;25(17):4023. 10.3390/molecules25174023. 32899230 10.3390/molecules25174023 PMC7570011

[36] Sharma N, Kumar J, Abedin MM, Sahoo D, Pandey A, Rai AK, et al. Metagenomics revealing molecular profiling of community structure and metabolic pathways in natural hot springs of the Sikkim Himalaya. BMC Microbiol. 2020;20(1):246. 10.1186/s12866-020-01923-3. 32778049 10.1186/s12866-020-01923-3 PMC7418396

[37] DeCastro M-E, Escuder-Rodríguez J-J, Becerra M, Rodríguez-Belmonte E, González-Siso M-I. Comparative Metagenomic Analysis of Two Hot Springs From Ourense (Northwestern Spain) and Others Worldwide. Front Microbiol. 2021;12. 10.3389/fmicb.2021.769065. 10.3389/fmicb.2021.769065 PMC8661477 34899652

[38] Allioux M, Yvenou S, Merkel A, Cozannet M, Aube J, Pommellec J, et al. A metagenomic insight into the microbiomes of geothermal springs in the Subantarctic Kerguelen Islands. Sci Rep. 2022;12(1):22243. 10.1038/s41598-022-26299-4. 36564496 10.1038/s41598-022-26299-4 PMC9789041

[39] Saini N, Aamir M, Singh VK, Deepak B, Mona S. Unveiling the microbial diversity and functional dynamics of Shiv Kund, Sohna hot spring, India through a shotgun metagenomics approach. Arch Microbiol. 2023;205(9):323. 10.1007/s00203-023-03664-z. 37651004 10.1007/s00203-023-03664-z

[40] Nagar S, Bharti M, Negi RK. Genome-resolved metagenomics revealed metal-resistance, geochemical cycles in a Himalayan hot spring. Appl Microbiol Biotechnol. 2023;107(10):3273–89. 10.1007/s00253-023-12503-6. 37052633 10.1007/s00253-023-12503-6

[41] Colman DR, Keller LM, Arteaga-Pozo E, Andrade-Barahona E, St Clair B, Shoemaker A, et al. Covariation of hot spring geochemistry with microbial genomic diversity, function, and evolution. Nat Commun. 2024;15(1):7506. 10.1038/s41467-024-51841-5. 39209850 10.1038/s41467-024-51841-5 PMC11362583

[42] Jaito N, Kaewsawat N, Phetlum S, Uengwetwanit T. Metagenomic discovery of lipases with predicted structural similarity to Candida antarctica lipase B. PLoS ONE. 2023;18(12):e0295397. 10.1371/journal.pone.0295397. 38055755 10.1371/journal.pone.0295397 PMC10699602

[43] Strazzulli A, Cobucci-Ponzano B, Iacono R, Giglio R, Maurelli L, Curci N, et al. Discovery of hyperstable carbohydrate-active enzymes through metagenomics of extreme environments. FEBS J. 2020;287(6):1116–37. 10.1111/febs.15080. 31595646 10.1111/febs.15080

[44] Knapik K, Becerra M, Gonzalez-Siso MI. Microbial diversity analysis and screening for novel xylanase enzymes from the sediment of the Lobios Hot Spring in Spain. Sci Rep. 2019;9(1):11195. 10.1038/s41598-019-47637-z. 31371784 10.1038/s41598-019-47637-z PMC6671963

[45] Castelan-Sanchez HG, Meza-Rodriguez PM, Carrillo E, Rios-Vazquez DI, Linan-Torres A, Batista-Garcia RA, et al. The Microbial Composition in Circumneutral Thermal Springs from Chignahuapan, Puebla, Mexico Reveals the Presence of Particular Sulfur-Oxidizing Bacterial and Viral Communities. Microorganisms. 2020;8(11). 10.3390/microorganisms8111677. 10.3390/microorganisms8111677 PMC7692377 33137872

[46] Alcorta J, Alarcon-Schumacher T, Salgado O, Diez B. Taxonomic Novelty and Distinctive Genomic Features of Hot Spring Cyanobacteria. Front Genet. 2020;11:568223. 10.3389/fgene.2020.568223. 33250920 10.3389/fgene.2020.568223 PMC7674949

[47] Ward LM, Idei A, Nakagawa M, Ueno Y, Fischer WW, McGlynn SE. Geochemical and Metagenomic Characterization of Jinata Onsen, a Proterozoic-Analog Hot Spring, Reveals Novel Microbial Diversity including Iron-Tolerant Phototrophs and Thermophilic Lithotrophs. Microbes Environ. 2019;34(3):278–92. 10.1264/jsme2.ME19017. 31413226 10.1264/jsme2.ME19017 PMC6759342

[48] Lin KH, Liao BY, Chang HW, Huang SW, Chang TY, Yang CY, et al. Metabolic characteristics of dominant microbes and key rare species from an acidic hot spring in Taiwan revealed by metagenomics. BMC Genomics. 2015;16:1029. 10.1186/s12864-015-2230-9. 26630941 10.1186/s12864-015-2230-9 PMC4668684

[49] Levy-Booth DJ, Hashimi A, Roccor R, Liu LY, Renneckar S, Eltis LD, et al. Genomics and metatranscriptomics of biogeochemical cycling and degradation of lignin-derived aromatic compounds in thermal swamp sediment. ISME J. 2021;15(3):879–93. 10.1038/s41396-020-00820-x. 33139871 10.1038/s41396-020-00820-x PMC8027834

[50] Lopez-Lopez O, Knapik K, Cerdan ME, Gonzalez-Siso MI. Metagenomics of an Alkaline Hot Spring in Galicia (Spain): Microbial Diversity Analysis and Screening for Novel Lipolytic Enzymes. Front Microbiol. 2015;6:1291. 10.3389/fmicb.2015.01291. 26635759 10.3389/fmicb.2015.01291 PMC4653306

[51] Tamburello G, Chiodini G, Ciotoli G, Procesi M, Rouwet D, Sandri L, et al. Global thermal spring distribution and relationship to endogenous and exogenous factors. Nat Commun. 2022;13(1):6378. 10.1038/s41467-022-34115-w. 36289217 10.1038/s41467-022-34115-w PMC9606316

[52] Biyada S, Merzouki M, Demcenko T, Vasiliauskiene D, Ivanec-Goranina R, Urbonavicius J, et al. Microbial community dynamics in the mesophilic and thermophilic phases of textile waste composting identified through next-generation sequencing. Sci Rep. 2021;11(1):23624. 10.1038/s41598-021-03191-1. 34880393 10.1038/s41598-021-03191-1 PMC8654937

[53] Buessecker S, Palmer M, Lai D, Dimapilis J, Mayali X, Mosier D, et al. An essential role for tungsten in the ecology and evolution of a previously uncultivated lineage of anaerobic, thermophilic Archaea. Nat Commun. 2022;13(1):3773. 10.1038/s41467-022-31452-8. 35773279 10.1038/s41467-022-31452-8 PMC9246946

[54] Hou W, Wang S, Dong H, Jiang H, Briggs BR, Peacock JP, et al. A comprehensive census of microbial diversity in hot springs of Tengchong, Yunnan Province China Using 16S rRNA gene Pyrosequencing. PLoS ONE. 2013;8(1):e53350. 10.1371/journal.pone.0053350. 23326417 10.1371/journal.pone.0053350 PMC3541193

[55] Inskeep WP, Klatt CG, Herrgard MJ, Jay ZJ, Rusch DB, Tringe SG, et al. Community Structure and Function of High-Temperature Chlorophototrophic Microbial Mats Inhabiting Diverse Geothermal Environments. Front Microbiol. 2013;4. 10.3389/fmicb.2013.00106. 10.3389/fmicb.2013.00106 PMC3669762 23761787

[56] Guo L, Wang G, Sheng Y, Sun X, Shi Z, Xu Q, et al. Temperature governs the distribution of hot spring microbial community in three hydrothermal fields, Eastern Tibetan Plateau Geothermal Belt, Western China. Sci Total Environ. 2020;720:137574. 10.1016/j.scitotenv.2020.137574. 32145630 10.1016/j.scitotenv.2020.137574

[57] Podar PT, Yang Z, Björnsdóttir SH, Podar M. Comparative Analysis of Microbial Diversity Across Temperature Gradients in Hot Springs From Yellowstone and Iceland. Front Microbiol. 2020;11. 10.3389/fmicb.2020.01625. 10.3389/fmicb.2020.01625 PMC7372906 32760379

[58] He Q, Wang S, Hou W, Feng K, Li F, Hai W, et al. Temperature and microbial interactions drive the deterministic assembly processes in sediments of hot springs. Sci Total Environ. 2021;772:145465. 10.1016/j.scitotenv.2021.145465. 33571767 10.1016/j.scitotenv.2021.145465

[59] Qi YL, Chen YT, Xie YG, Li YX, Rao YZ, Li MM, et al. Analysis of nearly 3000 archaeal genomes from terrestrial geothermal springs sheds light on interconnected biogeochemical processes. Nat Commun. 2024;15(1):4066. 10.1038/s41467-024-48498-5. 38744885 10.1038/s41467-024-48498-5 PMC11094006

[60] Jonson BD, Tsapekos P, Tahir Ashraf M, Jeppesen M, Ejbye Schmidt J, Bastidas-Oyanedel JR. Pilot-scale study of biomethanation in biological trickle bed reactors converting impure CO(2) from a Full-scale biogas plant. Bioresour Technol. 2022;365:128160. 10.1016/j.biortech.2022.128160. 36273766 10.1016/j.biortech.2022.128160

[61] Kim TW, Bae SS, Lee JW, Lee SM, Lee JH, Lee HS, et al. A biological process effective for the conversion of CO-containing industrial waste gas to acetate. Bioresour Technol. 2016;211:792–6. 10.1016/j.biortech.2016.04.038. 27106591 10.1016/j.biortech.2016.04.038

[62] Wang S, Hou W, Dong H, Jiang H, Huang L, Wu G, et al. Control of temperature on microbial community structure in hot springs of the Tibetan Plateau. PLoS ONE. 2013;8(5):e62901. 10.1371/journal.pone.0062901. 23667538 10.1371/journal.pone.0062901 PMC3647046

[63] Uribe-Lorío L, Brenes-Guillén L, Hernández-Ascencio W, Mora-Amador R, González G, Ramírez-Umaña CJ, et al. The influence of temperature and pH on bacterial community composition of microbial mats in hot springs from Costa Rica. MicrobiologyOpen. 2019;8(10):e893. 10.1002/mbo3.893. 31271524 10.1002/mbo3.893 PMC6813449

[64] Chiriac CM, Szekeres E, Rudi K, Baricz A, Hegedus A, Dragoş N, et al. Differences in Temperature and Water Chemistry Shape Distinct Diversity Patterns in Thermophilic Microbial Communities. Appl Environ Microbiol. 2017;83(21):e01363–17. 10.1128/AEM.01363-17. 28821552 10.1128/AEM.01363-17 PMC5648903

[65] Colman DR, Veach A, Stefansson A, Wurch L, Belisle BS, Podar PT, et al. Tectonic and geological setting influence hot spring microbiology. Environ Microbiol. 2023;25(11):2481–97. 10.1111/1462-2920.16472. 37553090 10.1111/1462-2920.16472

[66] Sriaporn C, Campbell KA, Van Kranendonk MJ, Handley KM. Bacterial and archaeal community distributions and cosmopolitanism across physicochemically diverse hot springs. ISME Commun. 2023;3(1):80. 10.1038/s43705-023-00291-z. 37596308 10.1038/s43705-023-00291-z PMC10439147

[67] Sharp CE, Brady AL, Sharp GH, Grasby SE, Stott MB, Dunfield PF. Humboldt’s spa: microbial diversity is controlled by temperature in geothermal environments. ISME J. 2014;8(6):1166–74. 10.1038/ismej.2013.237. 24430481 10.1038/ismej.2013.237 PMC4030231

[68] Miller SR, Strong AL, Jones KL, Ungerer MC. Bar-coded pyrosequencing reveals shared bacterial community properties along the temperature gradients of two alkaline hot springs in Yellowstone National Park. Appl Environ Microbiol. 2009;75(13):4565–72. 10.1128/AEM.02792-08. 19429553 10.1128/AEM.02792-08 PMC2704827

[69] Celińska E, Grajek W. Biotechnological production of 2, 3-butanediol—current state and prospects. Biotechnol Adv. 2009;27(6):715–25. 10.1016/j.biotechadv.2009.05.002. 19442714 10.1016/j.biotechadv.2009.05.002

[70] Raj T, Chandrasekhar K, Naresh Kumar A, Rajesh Banu J, Yoon J-J, Kant Bhatia S, et al. Recent advances in commercial biorefineries for lignocellulosic ethanol production: Current status, challenges and future perspectives. Bioresour Technol. 2022;344:126292. 10.1016/j.biortech.2021.126292. 34748984 10.1016/j.biortech.2021.126292

[71] Panaitescu DM, Frone AN, Chiulan I, Nicolae CA, Trusca R, Ghiurea M, et al. Role of bacterial cellulose and poly (3-hydroxyhexanoate-co-3-hydroxyoctanoate) in poly (3-hydroxybutyrate) blends and composites. Cellulose. 2018;25(10):5569–91. 10.1007/s10570-018-1980-3.

[72] Lee KY, Mooney DJ. Alginate: properties and biomedical applications. Prog Polym Sci. 2012;37(1):106–26. 10.1016/j.progpolymsci.2011.06.003. 22125349 10.1016/j.progpolymsci.2011.06.003 PMC3223967

[73] Bibi Z, Qader SA, Aman A. Calcium alginate matrix increases the stability and recycling capability of immobilized endo-beta-1,4-xylanase from Geobacillus stearothermophilus KIBGE-IB29. Extremophiles. 2015;19(4):819–27. 10.1007/s00792-015-0757-y. 26001519 10.1007/s00792-015-0757-y

[74] Velasco A, Acebo P, Gomez A, Schleissner C, Rodriguez P, Aparicio T, et al. Molecular characterization of the safracin biosynthetic pathway from Pseudomonas fluorescens A2-2: designing new cytotoxic compounds. Mol Microbiol. 2005;56(1):144–54. 10.1111/j.1365-2958.2004.04433.x. 15773985 10.1111/j.1365-2958.2004.04433.x

[75] Sato T, Yamawaki K. Cefiderocol: discovery, chemistry, and in vivo profiles of a novel siderophore cephalosporin. Clin Infect Dis. 2019;69(Supplement7):S538–43. 10.1093/cid/ciz826. 31724047 10.1093/cid/ciz826 PMC6853759

[76] Heilbronner S, Krismer B, Brötz-Oesterhelt H, Peschel A. The microbiome-shaping roles of bacteriocins. Nat Rev Microbiol. 2021;19(11):726–39. 10.1038/s41579-021-00569-w. 34075213 10.1038/s41579-021-00569-w

[77] Case RJ, Labbate M, Kjelleberg S. AHL-driven quorum-sensing circuits: their frequency and function among the Proteobacteria. ISME J. 2008;2(4):345–9. 10.1038/ismej.2008.13. 18273067 10.1038/ismej.2008.13

[78] Kuhlmann AU, Bremer E. Osmotically regulated synthesis of the compatible solute ectoine in Bacillus pasteurii and related Bacillus spp. Appl Environ Microbiol. 2002;68(2):772–83. 10.1128/AEM.68.2.772-783.2002. 11823218 10.1128/AEM.68.2.772-783.2002 PMC126723

[79] Avalos M, Garbeva P, Vader L, van Wezel GP, Dickschat JS, Ulanova D. Biosynthesis, evolution and ecology of microbial terpenoids. Nat Prod Rep. 2022;39(2):249–72. 10.1039/d1np00047k. 34612321 10.1039/d1np00047k

[80] Baginska S, Golognko A, Swislocka R, Lewandowski W. Monoterpenes as medicinal agents: Exploring the pharmaceutical potential of p-cymene, p-cymenene, and γ-terpinene. Acta Pol Pharm—Drug Res. 2023;80:879–92. 10.32383/appdr/178242.

[81] Mahizan NA, Yang S-K, Moo C-L, Song AA-L, Chong C-M, Chong C-W, et al. Terpene derivatives as a potential agent against antimicrobial resistance (AMR) pathogens. Molecules. 2019;24(14):2631. 10.3390/molecules24142631. 31330955 10.3390/molecules24142631 PMC6680751

[82] Ansari IA, Akhtar MS. Current insights on the role of terpenoids as anticancer agents: A perspective on cancer prevention and treatment. Natural Bio-active Compounds: Volume 2: Chemistry, Pharmacology and Health Care Practices. 2019:53–80; 10.1007/978-981-13-7205-6_3

[83] Gonzalez-Burgos E, Gomez-Serranillos M. Terpene compounds in nature: a review of their potential antioxidant activity. Curr Med Chem. 2012;19(31):5319–41. 10.2174/092986712803833335. 22963623 10.2174/092986712803833335

[84] Ajikumar PK, Tyo K, Carlsen S, Mucha O, Phon TH, Stephanopoulos G. Terpenoids: opportunities for biosynthesis of natural product drugs using engineered microorganisms. Mol Pharm. 2008;5(2):167–90. 10.1021/mp700151b. 18355030 10.1021/mp700151b

[85] Schröter M-A, Meyer S, Hahn MB, Solomun T, Sturm H, Kunte H-J. Ectoine protects DNA from damage by ionizing radiation. Sci Rep. 2017;7(1):15272. 10.1038/s41598-017-15512-4. 29127339 10.1038/s41598-017-15512-4 PMC5681641

[86] Kunte H-J, Lentzen G, Galinski E. Industrial production of the cell protectant ectoine: protection mechanisms, processes, and products. Curr Biotechnol. 2014;3(1):10–25. 10.2174/22115501113026660037.

[87] Strong P, Kalyuzhnaya M, Silverman J, Clarke W. A methanotroph-based biorefinery: potential scenarios for generating multiple products from a single fermentation. Bioresour Technol. 2016;215:314–23. 10.1016/j.biortech.2016.04.099. 27146469 10.1016/j.biortech.2016.04.099

[88] Dose B, Niehs SP, Scherlach K, Florez LV, Kaltenpoth M, Hertweck C. Unexpected bacterial origin of the antibiotic icosalide: two-tailed depsipeptide assembly in multifarious burkholderia symbionts. ACS Chem Biol. 2018;13(9):2414–20. 10.1021/acschembio.8b00600. 30160099 10.1021/acschembio.8b00600

[89] Vanittanakom N, Loeffler W, Koch U, Jung G. Fengycin-a novel antifungal lipopeptide antibiotic produced by Bacillus subtilis F-29-3. J Antibiot. 1986;39(7):888–901. 10.7164/antibiotics.39.888. 10.7164/antibiotics.39.888 3093430

[90] Villadsen NL, Jacobsen KM, Keiding UB, Weibel ET, Christiansen B, Vosegaard T, et al. Synthesis of ent-BE-43547A(1) reveals a potent hypoxia-selective anticancer agent and uncovers the biosynthetic origin of the APD-CLD natural products. Nat Chem. 2017;9(3):264–72. 10.1038/nchem.2657. 28221346 10.1038/nchem.2657

[91] Cochrane SA, Vederas JC. Lipopeptides from Bacillus and Paenibacillus spp.: a gold mine of antibiotic candidates. Med Res Rev. 2016;36(1):4–31. 10.1002/med.21321. 24866700 10.1002/med.21321

[92] Spraker JE, Schroeder FC, Wiemann P, Baccile JA, Venkatesh N, Schumacher J, et al. Conserved responses in a war of small molecules between a plant-pathogenic bacterium and fungi. mBio. 2018;9(3):2161–29. 10.1128/mBio.00820-18. 10.1128/mBio.00820-18 PMC5964348 29789359

[93] Malesevic M, Stanisavljevic N, Matijasevic D, Curcic J, Tasic V, Tasic S, et al. Metagenomic analysis of bacterial community and isolation of representative strains from Vranjska Banja hot spring, Serbia. Microb Ecol. 2023;86(4):2344–56. 10.1007/s00248-023-02242-6. 37222803 10.1007/s00248-023-02242-6

[94] Das S, Najar IN, Sherpa MT, Kumar S, Sharma P, Mondal K, et al. Baseline metagenome-assembled genome (MAG) data of Sikkim hot springs from Indian Himalayan geothermal belt (IHGB) showcasing its potential CAZymes, and sulfur-nitrogen metabolic activity. World J Microbiol Biotechnol. 2023;39(7):179. 10.1007/s11274-023-03631-2. 37133792 10.1007/s11274-023-03631-2

[95] Ruhl IA, Sheremet A, Smirnova AV, Sharp CE, Grasby SE, Strous M, et al. Microbial functional diversity correlates with species diversity along a temperature gradient. mSystems. 2022;7(1):e00991–21. 10.1128/msystems.00991-21. 35166562 10.1128/msystems.00991-21 PMC8845567

[96] Blin K, Wolf T, Chevrette MG, Lu X, Schwalen CJ, Kautsar SA, et al. antiSMASH 4.0-improvements in chemistry prediction and gene cluster boundary identification. Nucleic Acids Res. 2017;45(W1):W36–41. 10.1093/nar/gkx319. 28460038 10.1093/nar/gkx319 PMC5570095

[97] Medema MH, Blin K, Cimermancic P, de Jager V, Zakrzewski P, Fischbach MA, et al. antiSMASH: rapid identification, annotation and analysis of secondary metabolite biosynthesis gene clusters in bacterial and fungal genome sequences. Nucleic Acids Res. 2011;W339–46. 10.1093/nar/gkr466. 39(Web Server issue). 10.1093/nar/gkr466 PMC3125804 21672958

[98] Ramaswamy AV, Sorrels CM, Gerwick WH. Cloning and biochemical characterization of the hectochlorin biosynthetic gene cluster from the marine cyanobacterium Lyngbya majuscula. J Nat Prod. 2007;70(12):1977–86. 10.1021/np0704250. 18001088 10.1021/np0704250

[99] Teta R, Marteinsson VT, Longeon A, Klonowski AM, Groben R, Bourguet-Kondracki M-L, et al. Thermoactinoamide A, an antibiotic lipophilic cyclopeptide from the icelandic thermophilic bacterium Thermoactinomyces vulgaris. J Nat Prod. 2017;80(9):2530–5. 10.1021/acs.jnatprod.7b00560. 28841315 10.1021/acs.jnatprod.7b00560

[100] Schofield MM, Sherman DH. Meta-omic characterization of prokaryotic gene clusters for natural product biosynthesis. Curr Opin Biotechnol. 2013;24(6):1151–8. 10.1016/j.copbio.2013.05.001. 23731715 10.1016/j.copbio.2013.05.001 PMC3797859

[101] Crits-Christoph A, Diamond S, Butterfield CN, Thomas BC, Banfield JF. Novel soil bacteria possess diverse genes for secondary metabolite biosynthesis. Nature. 2018;558(7710):440–4. 10.1038/s41586-018-0207-y. 29899444 10.1038/s41586-018-0207-y

[102] Redl S, Sukumara S, Ploeger T, Wu L, Olshoj Jensen T, Nielsen AT, et al. Thermodynamics and economic feasibility of acetone production from syngas using the thermophilic production host Moorella thermoacetica. Biotechnol Biofuels. 2017;10:150. 10.1186/s13068-017-0827-8. 28616074 10.1186/s13068-017-0827-8 PMC5469130

[103] Bolger AM, Lohse M, Usadel B. Trimmomatic: a flexible trimmer for Illumina sequence data. Bioinformatics. 2014;30(15):2114–20. 10.1093/bioinformatics/btu170. 24695404 10.1093/bioinformatics/btu170 PMC4103590

[104] Menzel P, Ng KL, Krogh A. Fast and sensitive taxonomic classification for metagenomics with Kaiju. Nat Commun. 2016;7:11257. 10.1038/ncomms11257. 27071849 10.1038/ncomms11257 PMC4833860

[105] Nurk S, Meleshko D, Korobeynikov A, Pevzner PA. metaSPAdes: a new versatile metagenomic assembler. Genome Res. 2017;27(5):824–34. 10.1101/gr.213959.116. 28298430 10.1101/gr.213959.116 PMC5411777

[106] Langmead B, Salzberg SL. Fast gapped-read alignment with Bowtie 2. Nat Methods. 2012;9(4):357–9. 10.1038/nmeth.1923. 22388286 10.1038/nmeth.1923 PMC3322381

[107] Danecek P, Bonfield JK, Liddle J, Marshall J, Ohan V, Pollard MO, et al. Twelve years of SAMtools and BCFtools. Gigascience. 2021;10(2):giab008. 10.1093/gigascience/giab008. 33590861 10.1093/gigascience/giab008 PMC7931819

[108] Uritskiy GV, DiRuggiero J, Taylor J. MetaWRAP-a flexible pipeline for genome-resolved metagenomic data analysis. Microbiome. 2018;6(1):158. 10.1186/s40168-018-0541-1. 30219103 10.1186/s40168-018-0541-1 PMC6138922

[109] Chaumeil PA, Mussig AJ, Hugenholtz P, Parks DH. GTDB-Tk: a toolkit to classify genomes with the Genome Taxonomy Database. Bioinformatics. 2019;36(6):1925–7. 10.1093/bioinformatics/btz848. 31730192 10.1093/bioinformatics/btz848 PMC7703759

[110] Seemann T. Prokka: rapid prokaryotic genome annotation. Bioinformatics. 2014;30(14):2068–9. 10.1093/bioinformatics/btu153. 24642063 10.1093/bioinformatics/btu153

[111] Aramaki T, Blanc-Mathieu R, Endo H, Ohkubo K, Kanehisa M, Goto S, et al. KofamKOALA: KEGG Ortholog assignment based on profile HMM and adaptive score threshold. Bioinformatics. 2020;36(7):2251–2. 10.1093/bioinformatics/btz859. 31742321 10.1093/bioinformatics/btz859 PMC7141845

[112] Rodriguez RL, Konstantinidis KT. Nonpareil: a redundancy-based approach to assess the level of coverage in metagenomic datasets. Bioinformatics. 2014;30(5):629–35. 10.1093/bioinformatics/btt584. 24123672 10.1093/bioinformatics/btt584

[113] Rodriguez RL, Gunturu S, Tiedje JM, Cole JR, Konstantinidis KT. Nonpareil 3: Fast estimation of metagenomic coverage and sequence diversity. mSystems. 2018;3(3):e00039–18. 10.1128/mSystems.00039-18. 29657970 10.1128/mSystems.00039-18 PMC5893860

[114] Putri GH, Anders S, Pyl PT, Pimanda JE, Zanini F. Analysing high-throughput sequencing data in Python with HTSeq 2.0. Bioinformatics. 2022;38(10):2943–5. 10.1093/bioinformatics/btac166. 35561197 10.1093/bioinformatics/btac166 PMC9113351

[115] Wagner GP, Kin K, Lynch VJ. Measurement of mRNA abundance using RNA-seq data: RPKM measure is inconsistent among samples. Theory Biosci. 2012;131(4):281–5. 10.1007/s12064-012-0162-3. 22872506 10.1007/s12064-012-0162-3

[116] Pereira MB, Wallroth M, Jonsson V, Kristiansson E. Comparison of normalization methods for the analysis of metagenomic gene abundance data. BMC Genomics. 2018;19:1–17. 10.1186/s12864-018-4637-6. 29678163 10.1186/s12864-018-4637-6 PMC5910605

[117] Blin K, Shaw S, Kloosterman AM, Charlop-Powers Z, van Wezel GP, Medema MH, et al. antiSMASH 6.0: improving cluster detection and comparison capabilities. Nucleic Acids Res. 2021;49(W1):W29–35. 10.1093/nar/gkab335. 33978755 10.1093/nar/gkab335 PMC8262755

[118] Hyatt D, Chen G-L, LoCascio PF, Land ML, Larimer FW, Hauser LJ. Prodigal: prokaryotic gene recognition and translation initiation site identification. BMC Bioinformatics. 2010;11:1–11. 10.1186/1471-2105-11-119. 20211023 10.1186/1471-2105-11-119 PMC2848648

[119] Gurevich A, Saveliev V, Vyahhi N, Tesler G. QUAST: quality assessment tool for genome assemblies. Bioinformatics. 2013;29(8):1072–5. 10.1093/bioinformatics/btt086. 23422339 10.1093/bioinformatics/btt086 PMC3624806

[120] Virtanen P, Gommers R, Oliphant TE, Haberland M, Reddy T, Cournapeau D, et al. SciPy 1.0: fundamental algorithms for scientific computing in Python. Nat Methods. 2020;17(3):261–72. 10.1038/s41592-019-0686-2. 32015543 10.1038/s41592-019-0686-2 PMC7056644

[121] Pedregosa F, Varoquaux G, Gramfort A, Michel V, Thirion B, Grisel O, et al. Scikit-learn: Machine learning in Python. J Mach Learn Res. 2011;12:2825–30. 10.5555/1953048.2078195.

[122] Hunter JD, Matplotlib. A 2D graphics environment. Comput Sci Eng. 2007;9(03):90–5. 10.1109/MCSE.2007.55.

[123] Waskom ML. Seaborn: statistical data visualization. J Open Source Softw. 2021;6(60):3021. 10.21105/joss.03021.

[124] McKinney W. SciPy. 2010;445(1):51–6. 10.25080/Majora-92bf1922-00a. Data structures for statistical computing in Python.

