# Supplementary material for: Exploring the biotechnological potential of terrestrial hot spring microbiomes for CO2 utilisation
Source: Environ Microbiome. 2026 Mar 11;21:56. doi: 10.1186/s40793-026-00875-x (PMC13093976; doi:10.1186/s40793-026-00875-x)
Supplement: Supplementary file 1 — Supplementary Material 1. [file 40793_2026_875_MOESM1_ESM.docx]

**Exploring the biotechnological potential of hot spring microbiomes for CO_2_ utilisation**

Christopher E Stead^1^, Leanne Walker^1^, Carla Greco^1,2^, Toni Galloway^1,3^, Claire Cousins^3^, Franziska Nagel^1^, Rainer Breitling^1,4^, Eriko Takano^1,5^, Snædís Huld Björnsdóttir^6^, Sophie L Nixon^1^*.

^1^Manchester Institute of Biotechnology, University of Manchester, Manchester, UK

^2^Basecamp Research, London, UK

^3^School of Earth and Environmental Sciences, University of St Andrews, St Andrews, UK

^4^Bioinformatics Institute (BII), Agency for Science, Technology and Research (A*STAR), Singapore

^5^Singapore Integrative Biosystems and Engineering Research (SIBER) Strategic Research Translational Trust (SRTT), A*STAR, Singapore

^6^School of Engineering and Natural Sciences, University of Iceland, Iceland

*Corresponding author: sophie.nixon@manchester.ac.uk


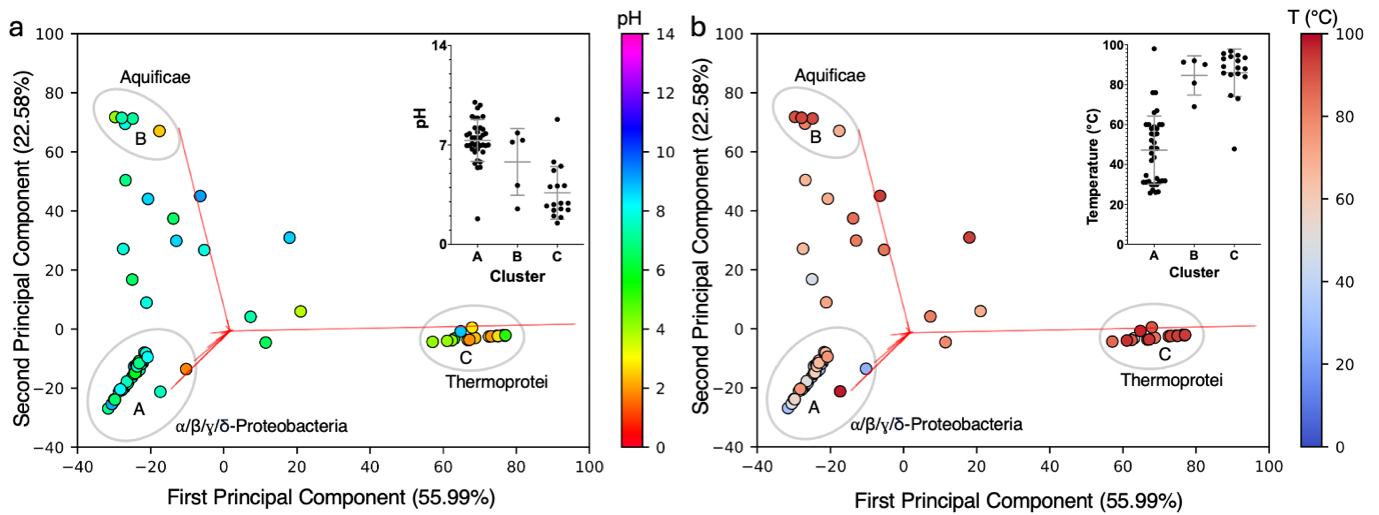


***Figure S1:*** *PCA of relative taxonomic abundance. Samples are coloured by physicochemical properties (pH in panel a; temperature in panel b), red lines indicate top 10 weighted loading vectors. Cluster A: Proteobacteria-dominated; Cluster B: Aquificae-dominated; Cluster C: Thermoprotei-dominated.*


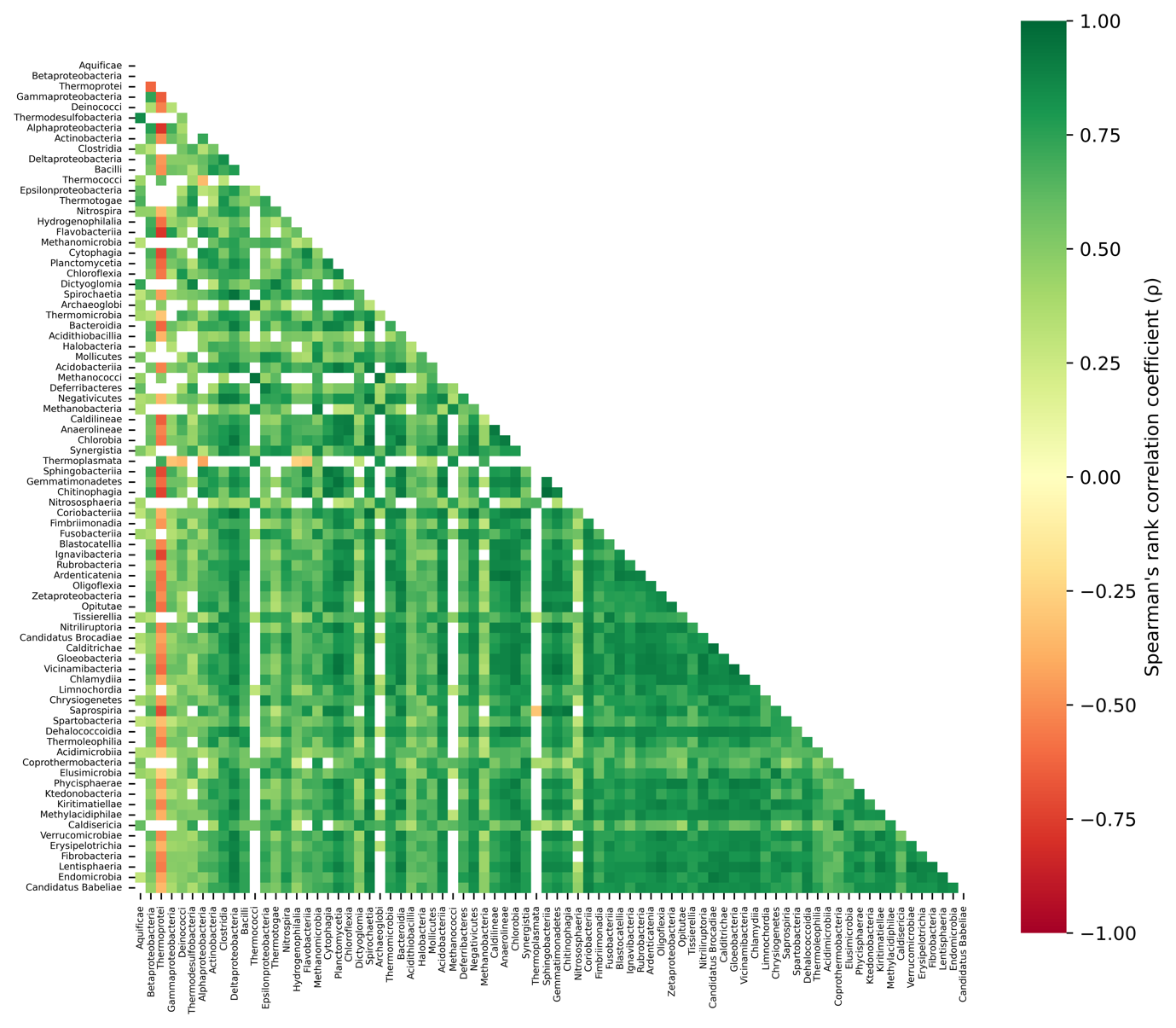


***Figure S2:*** *Spearman’s rank correlation matrix of microbial classes identified in all hot spring samples. Values with a P-values >0.01 excluded. The colour of the scale bar denotes the nature of the correlation with a perfect positive correlation (green), a perfect negative correlation (red) and no correlation (yellow).*

*
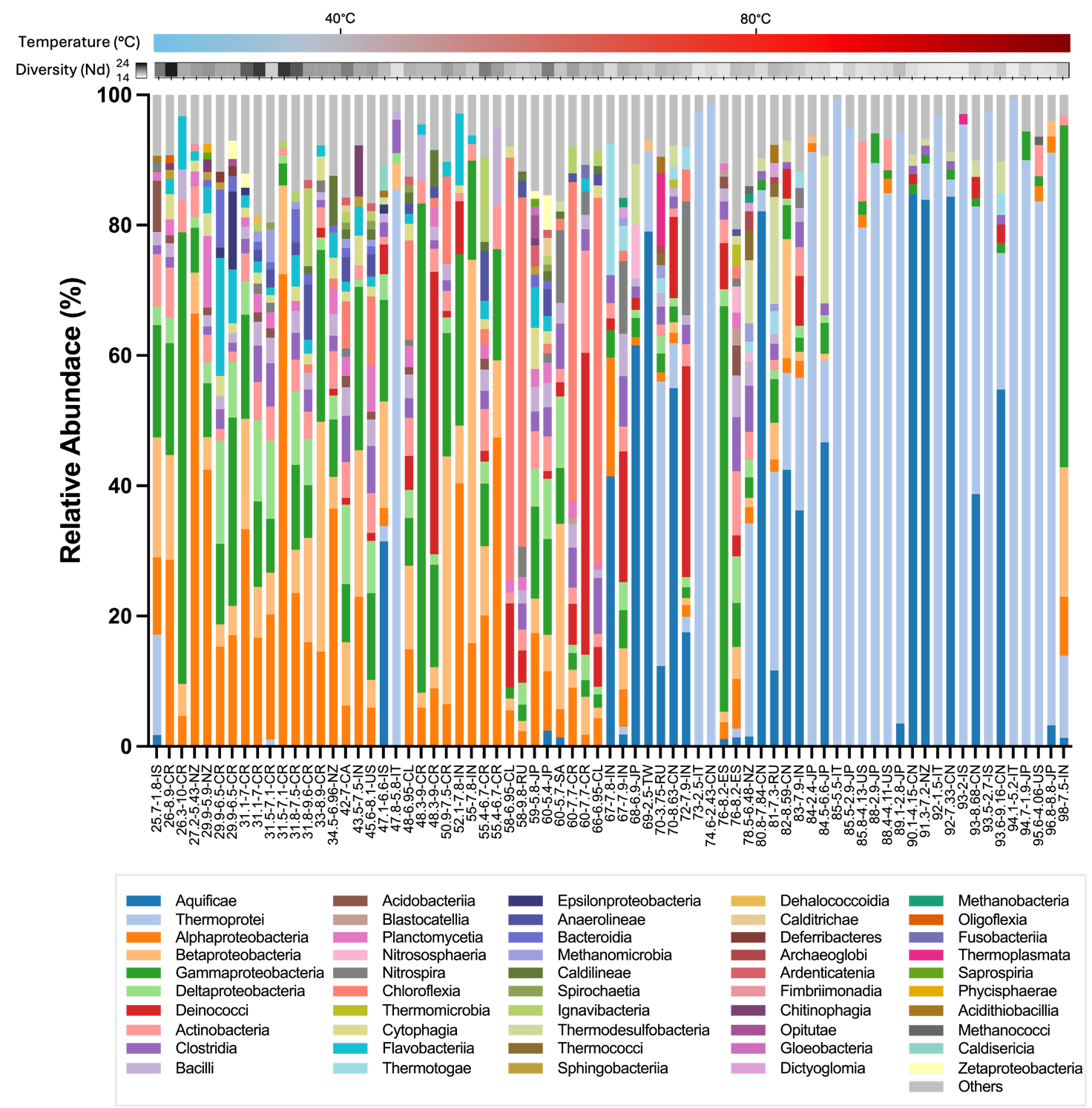
*

**Figure S3:** Relative abundance of taxa at the class level in hot springs. Taxa representing <1% of total reads are grouped as “Others”. Metagenomes are ordered by ascending temperature from left to right. Diversity (Nonpareil index, N_d_) is indicated above each bar. Sample IDs include temperature, pH, and country/region of origin (e.g., 47.1_6.6_IS). Country alpha-2 codes include: Canada, CA; Chile, CL; China, CN; Costa Rica, CR; Iceland, IS; India, IN; Italy, IT; Japan, JP; New Zealand, NZ; Russia, RU; South Africa, ZA; Spain, ES; Taiwan, TW; USA, US.


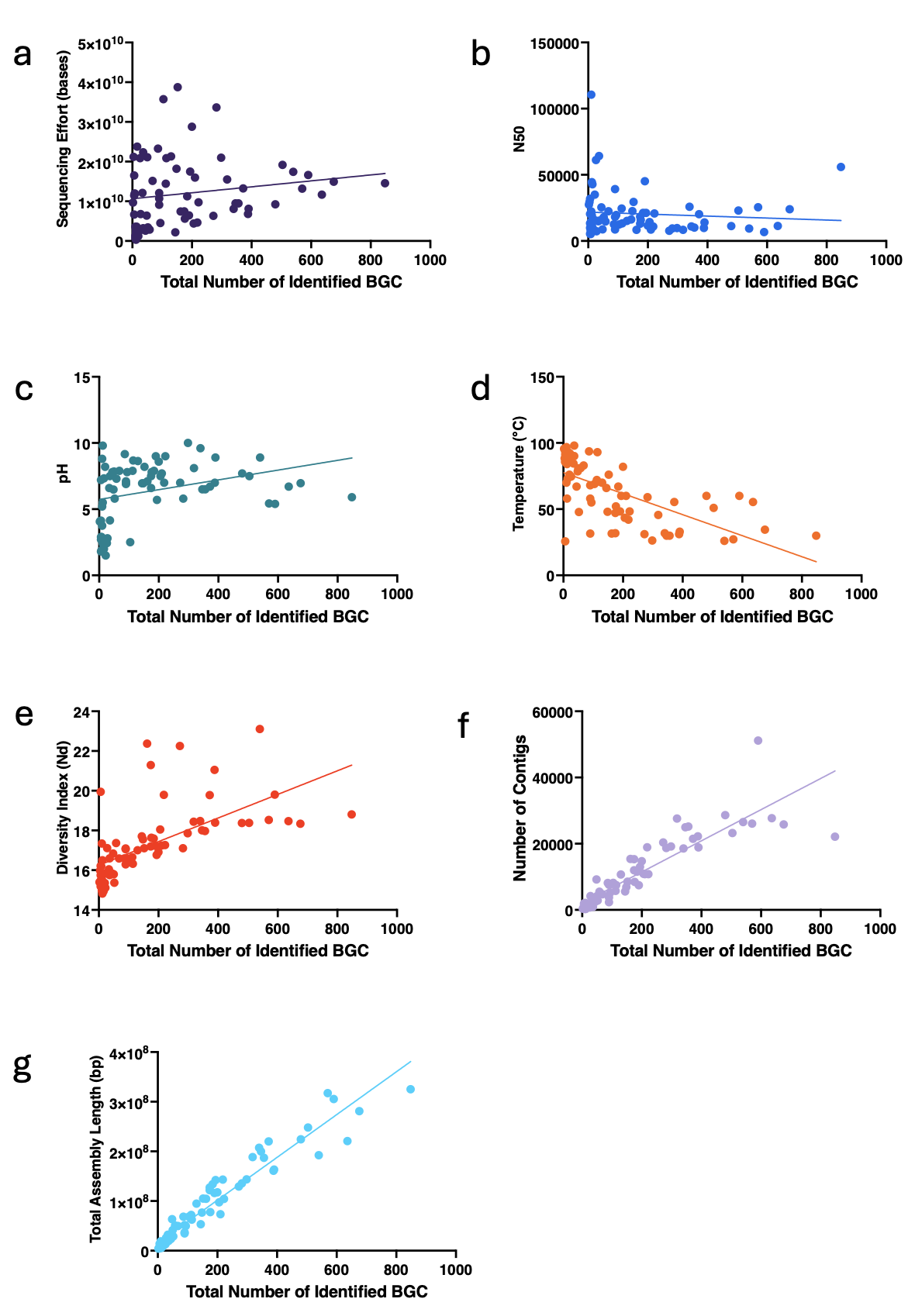


***Figure S4:*** *Scatter plots of identified biosynthetic gene clusters against potentially correlating influencing factors. a) Sequencing effort b) N50 c) pH d) Temperature e) Diversity index f) Number of contigs g) Total assembly length.*
